# Exploiting genetic polymorphisms in metabolic enzymes for rapid screening of *Leishmania infantum* genotypes

**DOI:** 10.1186/s13071-018-3143-7

**Published:** 2018-11-01

**Authors:** Marcello Ceccarelli, Aurora Diotallevi, Francesca Andreoni, Fabrizio Vitale, Luca Galluzzi, Mauro Magnani

**Affiliations:** 1 Department of Biomolecular Sciences, University of Urbino “Carlo Bo”, Urbino, PU Italy; 2 Istituto Zooprofilattico Sperimentale of Sicily “A Mirri”, Palermo, PA Italy

**Keywords:** *Leishmania infantum*, Isoenzymes, Zymodeme, MON, HRM, Malic enzyme, SNP

## Abstract

**Background:**

*Leishmania infantum* is the aetiological agent of visceral leishmaniasis (VL) and cutaneous leishmaniasis (CL). Numerous strains and/or zymodemes have been identified and characterized by multilocus enzyme electrophoresis (MLEE). MLEE is considered the reference method for *L. infantum* parasite typing and it is based upon enzyme electrophoretic mobility analysis from promastigote cultures. However, the MLEE technique is cumbersome, time-consuming and does not detect silent genetic mutations or nucleotide changes that give rise to amino acid changes that do not alter electrophoretic mobility. As a result of these difficulties, many DNA-based typing methods have been developed over the past few years. However, relative to the enzymes utilized in MLEE analysis, we observed a shortage of DNA sequences available in the GenBank database or an absolute lack of sequences belonging to specific zymodemes. The aims of the present study were to (i) implement the number of sequences coding for metabolic enzymes used in MLEE; (ii) identify polymorphisms that characterize *L. infantum* zymodemes most prevalent in the Mediterranean basin; and (iii) exploit these polymorphisms to develop a rapid screening test that would give results comparable with existing MLEE typing.

**Results:**

Partial sequences of seven metabolic enzyme genes (malic enzyme, 6-phosphogluconate dehydrogenase, mitochondrial isocitrate dehydrogenase, glucose-6-phosphate isomerase, glucose-6-phosphate dehydrogenase, phosphoglucomutase and mannose phosphate isomerase) were obtained from 11 *L. infantum* strains. The comparison of these sequences with those obtained from GenBank allowed for the identification of a few polymorphisms that could distinguish several zymodemes. In particular, the polymorphism 390T>G in the malic enzyme gene has been exploited to develop a high-resolution melt (HRM)-based assay to rapidly differentiate the genotype 390T, associated with zymodemes MON-1, MON-72 and MON-201, evidencing a partial agreement between genotyping results and MLEE. The assay has been successfully applied to *L. infantum* clinical isolates and clinical samples.

**Conclusions:**

A HRM-based assay for rapid identification of genotypes associated with the most common *L. infantum* zymodemes in the Mediterranean basin has been developed and its potential application in epidemiological research for *L. infantum* population screening, without parasite isolation and culturing, has been demonstrated.

**Electronic supplementary material:**

The online version of this article (10.1186/s13071-018-3143-7) contains supplementary material, which is available to authorized users.

## Background

The taxonomy of the genus *Leishmania* is complex and has been revised several times in light of parasite biology and biochemistry [1, 2]. Currently, the genus *Leishmania* includes four subgenera: *Leishmania*, *Viannia*, *Sauroleishmania* and *L. enriettii* complex. Each subgenus includes several species [1]. *Leishmania infantum*, the aetiological agent of visceral leishmaniasis (VL) and cutaneous leishmaniasis (CL), belongs to subgenus *Leishmania* [3].

Multilocus enzyme electrophoresis (MLEE) is the isoenzymatic analysis presently considered the reference method for parasite typing by the World Health Organization [4]. This technique, developed at the Centre for Leishmaniasis of Montpellier (France), is based on the electrophoretic mobility of several enzymes obtained from promastigote culture, as described by Rioux et al. [5]. Up to 15 enzymes can be considered in this analysis: malate dehydrogenase, malic enzyme, isocitrate dehydrogenase (NADP), phosphogluconate dehydrogenase, glucose-6-phosphate dehydrogenase, glutamate dehydrogenase, cytochrome-b5 reductase, purine-nucleoside phosphorylase 1 and 2, glutamic-oxaloacetic transaminase 1 and 2, phosphoglucomutase, fumarase, mannose-6-phosphate isomerase, and glucose-6-phosphate isomerase [6]. The comparison of isoenzyme mobility with a reference strain identified over 300 zymodemes, also termed MON.

Concerning *L. infantum*, 45 zymodemes have been found, 36 recognized as real zymodemes and 9 as variants [6]. In dogs in the Mediterranean area, 12 zymodemes have been found: MON-1, MON-24, MON-34, MON-72, MON-77, MON-80, MON-98, MON-105, MON-108, MON-199, MON-199 variant NP1130 and MON-281. With the exception of MON-105, these zymodemes have also all been found in humans [6]. Other zymodemes, such as MON-11, MON-27, MON-28, MON-29 MON-33 and MON-189, have been isolated only from humans [7]. *Leishmania infantum* MON-1 is the most frequent zymodeme in both humans and dogs in the Mediterranean basin [8] and in the New World [9]. It is present in 30 countries and represents about 70% of all identified strains. In particular, MON-1 represents 88% of *L. infantum* zymodemes in the south of France [10], 96.7% in Portugal [11] and 45–58% in Spain [12, 13]. Moreover, in HIV-infected subjects, MON-1 was found in about 73% of co-infections, while in immunocompetent patients it was found in about 90% of VL cases and 20% of CL cases [14]. Concerning canine infections in Italy, Gramiccia et al. [15] showed a high prevalence of MON-1 zymodeme (91%) in 497 canine clinical isolates, with the remaining percentage composed almost exclusively of MON-72 zymodeme. The homogeneity of the isoenzymes identified in the dog population does not fully reflect the isoenzyme diversity in humans; therefore, the role of canine population as the only reservoir for all *L. infantum* zymodemes remains unclear. In light of this epidemiological evidence, it is advisable that a more extensive typing of *L. infantum* be carried out. However, MLEE presents several disadvantages: it is time-consuming, technically demanding, and requires bulk cultures of parasites. Moreover, due to its inability to detect nucleotide substitutions that do not change the amino acid composition, its discriminatory power is poor. Therefore, molecular approaches such as multilocus microsatellite typing (MLMT) [16, 17] and multilocus sequence typing (MLST) [18–20] have been introduced for strain discrimination. MLST results were generally in agreement with MLEE, though increased resolution was obtained and some key discrepancies were found [18–21]. For example, *L. infantum* zymodeme MON-1 has been shown to be genetically heterogeneous. On the other hand, it was also shown that strains classified as different zymodemes can present the same genotype [18, 20]. However, the low number of typed strains and the lack of consensus on the marker genes, as well as the lack of MLST database for *Leishmania*, have hindered the implementation of MLST as a reference typing method [22]. Until now, most of the epidemiological data available in the literature is based on MLEE classification, and many DNA-based typing methods correlate data with existing MLEE classification [23, 24].

In this study, we explored the sequences of seven *L. infantum* genes encoding enzymes considered in MLEE analysis with the aim of (i) enriching the actual databases; (ii) identifying polymorphisms that characterize *L. infantum* zymodemes most prevalent in the Mediterranean basin; and (iii) developing a rapid screening test that would give results that could be associated with existing MLEE data, overcoming the drawbacks of this laborious technique.

## Methods

### *Leishmania infantum* DNA

*Leishmania infantum* strains or isolates used in this study are listed in Table 1. The Chelex-purified DNA from promastigotes of *L. infantum* strains or isolates was obtained from the National Reference Center for Leishmaniasis (C.Re.Na.L.), Istituto Zooprofilattico Sperimentale della Sicilia (Palermo, Italy). The DNA was quantified using a Qubit fluorometer (Life Technologies, Carlsbad, USA).

**Table 1:** *Leishmania infantum* strains/isolates used in this study

Species	Strain or clinical isolate	Zymodeme
*L. infantum*	MHOM/TN/80/IPT1	MON-1
*L. infantum*	MHOM/FR/78/LEM75	MON-1
*L. infantum*	Clinical isolate V2921	MON-1
*L. infantum*	Clinical isolate 31U	MON-1
*L. infantum*	Clinical isolate 49U	MON-1
*L. infantum*	Clinical isolate 10816	MON-1
*L. infantum*	Clinical isolate 791	MON-1
*L. infantum*	MHOM/DZ/82/LIPA59	MON-24
*L. infantum*	MHOM/ES/81/BCN1	MON-29
*L. infantum*	MHOM/IT/86/ISS218	MON-72
*L. infantum*	MHOM/IT/93/ISS822	MON-201
*L. infantum*	Isolate 1	Not determined
*L. infantum*	Isolate 2	Not determined

### Identification of molecular targets and PCR amplification

A preliminary *in silico* analysis was performed in the GenBank sequence database to search for genes encoding enzymes used in MLEE for *L. infantum*. These genes were selected not only to gain information on genetic diversity, but also to connect the genotyping results to the existing MLEE data. Six genes were initially selected, based on previous studies performed on *L. infantum* that allowed the retrieval of available polymorphic sequences from GenBank, i.e. malic enzyme (*me*), 6-phosphogluconate dehydrogenase (*pgd*), mitochondrial isocitrate dehydrogenase (*icd*), glucose-6-phosphate isomerase (*gpi*), glucose-6-phosphate dehydrogenase (*g6pdh*) and mannose phosphate isomerase (*mpi*). Moreover, the phosphoglucomutase (*pgm*) gene was also taken into consideration in an attempt to enrich the existing database. Primers were designed with Primer-BLAST [25] using *L. infantum* MHOM/FR/78/LEM75 or MCAN/AR/10/MDP1 sequences as reference. The primers are listed in Table 2 and their position on the seven target genes are shown in Additional file 1: Figure S1.

**Table 2:** Primers used in this study

Target gene	GenBank ID	Forward primer (5'-3')	Reverse primer (5'-3')	Amplicon length (bp)
Malic enzyme^a^	DQ449701.1	GAGCCGATCAACCGCTATCA	TCTTTCTTCATCCCGGCCTC	773
Malic enzyme^b^	DQ449701.1	GGTGTGGCGGAGAGCATC	TTGTTGCCTTGCGATGGTTG	458
Malic enzyme^c^	DQ449701.1	TCAGAACCTTCGCAAGACGA	CACTTGCCGATGCTGATGC	111
Phosphogluconate dehydrogenase	AM157139.1	TTCGGCTTCGACAACGATCA	CGAGGGAAGTTGGGGAATG	306
Mitochondrial isocitrate dehydrogenase	DQ449672.1	CTCCAGCACCAACGTCTACC	TACATGCGCTGGAAGGTCTG	708
Glucose-6-phosphate isomerase	AJ620617.1	CATTCACCAGGGCACCAAGA	TGATCGGAGACGATGTTGCC	426
Glucose-6-phosphate dehydrogenase	DQ449770.1	ATGTCGGAAGAGCAGTCTCA	CTCCTTCCCGAGGTAGTGGT	765
Phosphoglucomutase	KJ643214.1	GGAGACGGTTAAGATTACGCAC	TATGCTTCATCGCGGGGTT	413
Mannose phosphate isomerase	DQ449737.1	TTTGCGGAGTTGTGGGTAGG	CTCGCTGCTCTTCTTCTCGT	864

^a^5' region

^b^3' region

^c^Internal region

Conventional PCR was performed on a total volume of 50 μl with 1–2 μl of template, 200 μM dNTP, 2.5 mM MgCl_2_, 200 nM of each primer and 1 unit of Hot-Rescue DNA polymerase (Diatheva s.r.l., Fano, Italy). The amplification was performed in a PCR GeneAmp 2700 thermocycler (Applied Biosystems, Foster City, USA). The thermal cycling profile was as follows: 94 °C for 7 min; followed by 35 cycles at 94 °C for 30 s, 60 °C for 20 s or 60 s (depending on amplicon length) and 72 °C for 20 s; with a final extension at 72 °C for 5 min. Each sample was amplified in duplicate. Amplified fragments were analyzed by agarose gel electrophoresis and visualized with GelRed DNA stain (Biotium, Fremont, USA). The gels were visualized under UV light using a gel doc apparatus (Bio-Rad, Hercules, USA). A 100-bp double-stranded DNA ladder or ΦX174 DNA/BSU/ HaeIII/marker 9 (MBI Fermentas, Waltham, USA) was included on the gels as a size standard.

### PCR product sequencing and phylogenetic analysis

The amplification products of *me*, *pgd*, *icd*, *gpi*, *g6pdh*, *mpi* and *pgm* genes for all the strains/isolates are indicated in Table 1 (with the exception of isolate 1 and isolate 2) were purified using a Minelute PCR purification kit (Qiagen, Hilden, Germany) and directly sequenced. DNA sequencing was performed using the BigDye Terminator v. 1.1 Cycle Sequencing Kit on an ABI PRISM 310 Genetic Analyzer (Applied Biosystems). Bases with Phred values < 20 were checked by visual analysis of electropherograms and aligned with BioEdit Sequence Alignment Editor [26] using default options. Heterozygosity was considered to be present when direct sequencing of PCR product yielded a similar peak at the same site. The sequences were deposited in GenBank with the following accession numbers: MF375413-MF375423 (*me*), MF280205-MF280215 (*pgd*), MF347625-MF347635 (*icd*), MF288905-MF288915 (*gpi*), MF479731-MF479741 (*g6pdh*), MF462101-MF462111 (*mpi*) and MF347614-MF347624 (*pgm*).

For each housekeeping gene, allele numbers were assigned to unique sequences and a genotype was determined as the combination of the six genes selected for analysis (Additional file 2: Table S1). In total, 54 isolates/strains belonging to 22 different MON, were analyzed; 23 of them had sequences available for *me*, *pgd*, *icd*, *gpi*, *g6pdh* and *mpi* genes. For each of these isolates/strains, we concatenated those sequences to obtain a 3578 bp-long sequence. A maximum likelihood tree was constructed from the concatenated nucleotide dataset using PhyML 3.0 [27]. The best-fit substitution model was determined by the Akaike information criterion (AIC) using Smart Model Selection [28]. The optimal model of evolution was GTR+I with proportion of invariable sites of 0.989 and one category for substitution rate. Bootstrap values were calculated from 100 replications. The tree was visualized using iTOL [29].

### Real-time PCR and high resolution melting (HRM) analysis

A 111 bp internal region of the *me* gene encompassing the single nucleotide polymorphism (SNP) 390T>G was amplified with primers listed in Table 2. Real-time PCR (named qPCR-MEint) was carried out in a 25 μl volume with 1 μl template DNA and SYBR green PCR master mix (Diatheva s.r.l.) containing 1U Taq Polymerase and 200 nM of each primer. The PCR reactions were performed in a Rotor-Gene 6000 instrument (Corbett Life Science, Mortlake, Australia). The amplification profile was: 94 °C for 10 min; followed by 33 cycles at 94 °C for 30 s, 60 °C for 20 s and 72 °C for 20 s. The reactions were performed in duplicate. After amplification, the high-resolution melting (HRM) analysis was performed over the range 85–95 °C, rising by 0.1 °C/s and waiting for 2 s at each temperature. Raw HRM curves were normalized by the Rotorgene 6000 v.1.7 software. Difference graphs of the normalized curves were obtained using MHOM/FR/78/LEM75 and MHOM/DZ/82/LIPA59 strains as reference curves for genotypes 390T and 390G, respectively.

The qPCR-MEint and HRM analysis was also tested using DNA extracted from 4 clinical samples (whole blood, buffy coat, conjunctival swabs, bone marrow aspirate) as described previously [30, 31]. To ensure adequate sensitivity with these samples, a pre-amplification step (10 cycles) was performed and 2 μl of pre-amplified mixture were used as template in the qPCR described above.

## Results

### Sequence analyses

The *L. infantum* isolates/strains gave single products of expected size with the primer pairs listed in Table 2. The PCR products were sequenced and the sequences were aligned with those obtained from GenBank. Partial sequences of *me*, *pgd*, *icd*, *gpi*, *g6pdh*, *mpi* and *pgm* genes were obtained from 11 strains/isolates of *L. infantum*. Seven represented MON-1 zymodeme and the remaining four represented MON-24, MON-29, MON-72 and MON-201 (Table 1). Notably, the following *L. infantum* sequences from zymodemes not previously reported in the databases were obtained: *me* sequences from MON-24, MON-72 and MON-201; a *pgd* sequence from MON-201; *icd* sequences from MON-24, MON-72 and MON-201; *gpi* sequences from MON-24, MON-72 and MON-201; *g6pdh* sequences from MON-24, MON-72 and MON-201; *mpi* sequences from MON-24, MON-72 and MON-201; and *pgm* sequences from MON-1, MON-24, MON-29, MON-72 and MON-201.

Concerning the *me* gene, 13 SNPs were evidenced (Table 3). Nine were silent and four changed the amino acid sequence (V59M; I133S; V330I; E563E/D). These data confirmed previously reported findings obtained with *L. donovani* complex [19]. All sequences from MON-1 strains were identical, except for the MHOM/TN/80/IPT1 strain. Notably, the genotype heterogeneity of this strain was previously confirmed on the *gpi* sequence [18]. It is also noteworthy that the two MON-29 strains (MHOM/ES/82/BCN1 and MHOM/FR/1996/LEM3249) showed differences at nucleotide positions 327, 329 and 507, confirming the higher discriminatory power of a sequencing approach compared to MLEE. The *pgd* gene sequence showed only the silent SNP 678A>G in 11 strains (Table 4). Concerning the *icd* gene, it is of relevance that two silent SNPs (204T>C, 369T>C) were evidenced in strain MHOM/DZ/82/LIPA59 (MON-24) and three silent SNPs (150G>A, 369T>C, 1038C>A) in strain MHOM/SD/1997/LEM3472 (MON-267) (Table 5). The polymorphisms 204T>C and 1038C>A appeared unique for MON-24 and MON-267, respectively. The *gpi* gene sequence showed 1503G>T/K and 1831G>A/R SNPs in 4 and 5 strains, respectively (Table 6). The *g6pdh* gene showed 8 SNPs (Table 7). The *mpi* gene showed 5 SNPs (Table 8), with SNP 486G>A unique for MON-136. Finally, concerning the *pgm* gene, no differences were found among the zymodemes examined, including the two sequences available in the database.

**Table 3:** Polymorphisms in malic enzyme (*me*) gene

Zymodeme	Strain/isolate	GenBank ID	106	204	327	329	390	507	586	762	843	919	1182	1287	1620
MON-1	MHOM/FR/78/LEM75	DQ449701.1	G	C	C	T	T	T	C	C	C	G	A	C	G
MON-1	MHOM/ES/1993/PM1	DQ449703.1	**.**	**.**	**.**	**.**	**.**	**.**	**.**	**.**	**.**	**.**	**.**	**.**	**.**
MON-1	MHOM/FR/1995/LPN114	DQ449702.1	**.**	**.**	**.**	**.**	**.**	**.**	**.**	**.**	**.**	**.**	**.**	**.**	**.**
MON-1	MHOM/PT/2000/IMT260	DQ449706.1	**.**	**.**	**.**	**.**	**.**	**.**	**.**	**.**	**.**	**.**	**.**	**.**	**.**
MON-1	MHOM/FR/1997/LSL29	DQ449704.1	**.**	**.**	**.**	**.**	**.**	**.**	**.**	**.**	**.**	**.**	**.**	**.**	**.**
MON-1	MHOM/ES/1986/BCN16	DQ449705.1	**.**	**.**	**.**	**.**	**.**	**.**	**.**	**.**	**.**	**.**	**.**	**.**	**.**
MON-1	MHOM/BL/67/ITMAP263	KU175196.1	**.**	**.**	**.**	**.**	**.**	**.**	**.**	**.**	**.**	**.**	**.**	**.**	–
**MON-1**	**MHOM/TN/80/IPT1**	**MF375413**	–	**.**	**.**	**.**	G	**.**	**.**	**.**	**.**	–	–	–	–
**MON-1**	**MHOM/FR/78/LEM75**	**MF375414**	–	**.**	**.**	**.**	**.**	**.**	**.**	**.**	**.**	–	–	–	–
**MON-1**	**Isolate 31U**	**MF375415**	–	**.**	**.**	**.**	**.**	**.**	**.**	**.**	**.**	–	–	–	–
**MON-1**	**Isolate 49u**	**MF375416**	–	**.**	**.**	**.**	**.**	**.**	**–**	**.**	**.**	–	–	–	–
**MON-1**	**Isolate 10816**	**MF375417**	–	**.**	**.**	**.**	**.**	**.**	**.**	**.**	**.**	–	–	–	–
**MON-1**	**Isolate 791**	**MF375418**	–	**.**	**.**	**.**	**.**	**.**	**.**	**.**	**.**	–	–	–	–
**MON-1**	**Isolate V2921**	**MF375419**	–	–	–	–	–	–	–	**.**	**.**	–	–	–	–
MON-11	MHOM/FR/1980/LEM189	DQ449714.1	**.**	**.**	**.**	**.**	G	**.**	**.**	**.**	**.**	**.**	**.**	**.**	**.**
**MON-24**	**MHOM/DZ/82/LIPA59**	**MF375420**	–	T	**.**	G	G	G	**.**	**.**	T	–	–	–	–
**MON-29**	**MHOM/ES/82/BCN1**	**MF375421**	–	**.**	M	G	G	G	**.**	**.**	**.**	–	–	–	–
MON-29	MHOM/FR/1996/LEM3249	DQ449707.1	**.**	**.**	**.**	**.**	G	**.**	**.**	**.**	**.**	**.**	**.**	**.**	**.**
**MON-72**	**MHOM/IT/86/ISS218**	**MF375422**	–	**.**	**.**	**.**	**.**	**.**	**.**	**.**	**.**	**.**	**.**	**.**	–
MON-78	MHOM/MT/1985/BUCK	DQ449715.1	–	**.**	**.**	G	G	G	**.**	**.**	**.**	A	C	T	**.**
MON-81	MHOM/SD/1962/3S^a^	DQ449718.1	–	**.**	**.**	G	G	G	A	G	**.**	A	**.**	**.**	**.**
MON-98	MHOM/GR/2001/GH6	DQ449728.1	**.**	**.**	**.**	**.**	G	**.**	**.**	**.**	**.**	**.**	**.**	**.**	**.**
MON-98	MCAN/GR/2001/GD8	DQ449729.1	**.**	**.**	**.**	**.**	G	**.**	**.**	**.**	**.**	**.**	**.**	**.**	**.**
MON-98	MHOM/GR/2003/GH15	DQ449730.1	**.**	**.**	**.**	**.**	G	**.**	**.**	**.**	**.**	**.**	**.**	**.**	**.**
MON-98	MHOM/GR/2003/GH16	DQ449731.1	**.**	**.**	**.**	**.**	G	**.**	**.**	**.**	**.**	**.**	**.**	**.**	**.**
MON-98	MHOM/GR/2003/GH18	DQ449732.1	**.**	**.**	**.**	**.**	G	**.**	**.**	**.**	**.**	**.**	**.**	**.**	**.**
MON-98	MHOM/GR/2003/GH20	DQ449733.1	**.**	**.**	**.**	**.**	G	**.**	**.**	**.**	**.**	**.**	**.**	**.**	**.**
MON-98	MHOM/GR/2004/GD17	DQ449734.1	**.**	**.**	**.**	**.**	G	**.**	**.**	**.**	**.**	**.**	**.**	**.**	**.**
MON-183	MHOM/ES/1991/LEM2298	DQ449708.1	**.**	**.**	**.**	**.**	G	**.**	**.**	**.**	**.**	**.**	**.**	**.**	**.**
MON-188	MHOM/IT/1993/ISS800	DQ449722.1	**.**	T	**.**	G	G	G	**.**	**.**	T	A	**.**	**.**	**.**
MON-198	MHOM/ES/1988/LLM175	DQ449719.1	**.**	**.**	**.**	**.**	G	**.**	**.**	**.**	**.**	**.**	**.**	**.**	**.**
MON-199	MHOM/ES/1992/LLM373	DQ449720.1	A	**.**	**.**	G	G	G	**.**	**.**	**.**	**.**	C	**.**	**.**
**MON-201**	**MHOM/IT/93/ISS822**	**MF375423**	**.**	**.**	**.**	**.**	**.**	**.**	**.**	**.**	**.**	**.**	**.**	**.**	–
MON-228	MHOM/IT/1994/ISS1036	DQ449721.1	**.**	**.**	**.**	**.**	G	**.**	**.**	**.**	**.**	**.**	**.**	**.**	S
MON-267	MHOM/SD/1997/LEM3472^a^	DQ449723.1	**.**	**.**	**.**	G	G	G	**.**	**.**	**.**	A	**.**	**.**	**.**
MON-309	ITOB/TR/2005/CUK2	EU545253.1	**.**	T	**.**	K	G	G	**.**	**.**	Y	A	**.**	**.**	**.**
MON-309	MHOM/TR/2005/CUK1	EU545252.1	**.**	T	**.**	G	G	G	**.**	**.**	T	A	**.**	**.**	**.**
ni	ITOB/TR/2007/CUK10	EU545254.1	**.**	T	**.**	K	G	G	**.**	**.**	Y	A	**.**	**.**	**.**
non-MON-1	MHOM/TR/2000/OG-VL	EU545255.1	**.**	**.**	**.**	**.**	G	**.**	**.**	**.**	**.**	**.**	**.**	**.**	**.**
ni	Isolate RRR-B	DQ449727.1	**.**	**.**	**.**	**.**	G	**.**	**.**	**.**	**.**	**.**	**.**	**.**	**.**

Zymodeme sequences obtained in this study are in bold

*Abbreviation*: *ni* not indicated

*Key*: **.** consensus sequence, – sequence not available

^a^Initially assigned to *L. infantum* and successively designated *L. donovani* [18]

**Table 4:** Polymorphisms in 6-phosphogluconate dehydrogenase (*pgd*)

Zymodeme	Strain/isolate	GenBank ID	194	678	747
MON-1	MHOM/FR/78/LEM75	AM157139.1	C	A	C
MON-1	MHOM/PT/2000/IMT260 (LEM3975)	AM157144.1	**.**	**.**	**.**
MON-1	MHOM/ES/1986/BCN16 (LEM1078)	AM157143.1	**.**	**.**	**.**
MON-1	MHOM/FR/1997/LSL29 (LEM3420)	AM157142.1	**.**	**.**	**.**
MON-1	MHOM/ES/1993/PM1 (LEM2608)	AM157141.1	**.**	**.**	**.**
MON-1	MHOM/FR/1995/LPN114 (LEM3001)	AM157140.1	**.**	**.**	**.**
MON-1	MHOM/TN/1980/IPT1	AM157736.1	**.**	**.**	**.**
**MON-1**	**MHOM/TN/1980/IPT1**	**MF280205**	–	**.**	**.**
**MON-1**	**Isolate 31u**	**MF280206**	–	**.**	**.**
**MON-1**	**Isolate 49u**	**MF280207**	–	**.**	**.**
**MON-1**	**Isolate 791**	**MF280208**	–	**.**	**.**
**MON-1**	**Isolate 10816**	**MF280209**	–	**.**	**.**
**MON-1**	**MHOM/FR/78/LEM75**	**MF2802010**	–	**.**	**.**
**MON-1**	**Isolate V2921**	**MF2802011**	–	**.**	**.**
MON-11	MHOM/FR/1980/LEM189	AM157151.1	**.**	G	**.**
**MON-24**	**MHOM/DZ/82/LIPA59**	**MF2802012**	–	G	**.**
MON-24	IARI/PT/1989/IMT171	AM157137.1	**.**	G	**.**
MON-27	MHOM/IT/1979/Francesca	AM157138.1	**.**	**.**	**.**
**MON-29**	**MHOM/ES/82/BCN1**	**MF2802013**	–	G	**.**
MON-29	MHOM/FR/1996/LEM3249	AM157145.1	**.**	G	**.**
MON-34	MHOM/CN/1980/A	AM157136.1	**.**	G	**.**
**MON-72**	**MHOM/IT/86/ISS218**	**MF2802014**	–	**.**	**.**
MON-78	MHOM/MT/1985/BUCK	AM157152.1	**.**	**.**	**.**
MON-81	MHOM/SD/1962/3S^a^	AM157155.1	T	G	T
MON-183	MHOM/ES/1991/LEM2298	AM157146.1	**.**	G	**.**
MON-188	MHOM/IT/1993/ISS800	AM157159.1	**.**	G	**.**
MON-198	MHOM/ES/1988/LLM175	AM157156.1	**.**	G	**.**
MON-199	MHOM/ES/1992/LLM373	AM157157.1	**.**	G	**.**
**MON-201**	**MHOM/IT/93/ISS822**	**MF2802015**	–	**.**	**.**
MON-228	MHOM/IT/1994/ISS1036	AM157158.1	**.**	G	**.**
MON-267	MHOM/SD/1997/LEM3472^a^	AM157160.1	T	G	T

Zymodeme sequences obtained in this study are in bold

*Key*: **.** consensus sequence, – sequence not available

^a^Initially assigned to *L. infantum* and successively designated *L. donovani* [18]

**Table 5:** Polymorphisms in mitochondrial isocitrate dehydrogenase (*icd*)

Zymodeme	Strain/isolate	GenBank ID	150	204	369	1038
MON-1	MHOM/FR/78/LEM75	DQ449672.1	G	T	T	C
MON-1	MHOM/ES/1986/BCN16	DQ449676.1	**.**	**.**	**.**	**.**
MON-1	MHOM/FR/1997/LSL29	DQ449675.1	**.**	**.**	**.**	**.**
MON-1	MHOM/ES/1993/PM1	DQ449674.1	**.**	**.**	**.**	**.**
MON-1	MHOM/FR/1995/LPN114	DQ449673.1|	**.**	**.**	**.**	**.**
MON-1	MHOM/PT/2000/IMT260	DQ449677.1	**.**	**.**	**.**	**.**
**MON-1**	**MHOM/TN/1980/IPT1**	**MF347625**	**.**	**.**	**.**	–
**MON-1**	**MHOM/FR/78/LEM75**	**MF347626**	**.**	**.**	**.**	–
**MON-1**	**Isolate 31u**	**MF347627**	**.**	**.**	**.**	–
**MON-1**	**Isolate 49u**	**MF347628**	**.**	**.**	**.**	–
**MON-1**	**Isolate 791**	**MF347629**	**.**	**.**	**.**	–
**MON-1**	**Isolate 10816**	**MF347630**	**.**	**.**	**.**	–
**MON-1**	**Isolate V2921**	**MF347631**	**.**	**.**	**.**	–
MON-11	MHOM/FR/1980/LEM189	DQ449685.1	**.**	**.**	C	**.**
**MON-24**	**MHOM/DZ/82/LIPA59**	**MF347632**	**.**	C	C	–
**MON-29**	**MHOM/ES/82/BCN1**	**MF347633**	**.**	**.**	**.**	–
MON-29	MHOM/FR/1996/LEM3249	DQ449678.1	**.**	**.**	C	**.**
**MON-72**	**MHOM/IT/86/ISS218**	**MF347634**	**.**	**.**	**.**	–
MON-78	MHOM/MT/1985/BUCK	DQ449686.1	**.**	**.**	C	**.**
MON-81	MHOM/SD/1962/3S^a^	DQ449689.1	A	**.**	C	**.**
MON-183	MHOM/ES/1991/LEM2298	DQ449679.1	**.**	**.**	**.**	**.**
MON-188	MHOM/IT/1993/ISS800	DQ449693.1	**.**	**.**	C	**.**
MON-198	MHOM/ES/1988/LLM175	DQ449690.1	**.**	**.**	**.**	**.**
MON-199	MHOM/ES/1992/LLM373	DQ449691.1	**.**	**.**	**.**	**.**
**MON-201**	**MHOM/IT/93/ISS822**	**MF347635**	**.**	**.**	**.**	–
MON-228	MHOM/IT/1994/ISS1036	DQ449692.1	**.**	**.**	C	**.**
MON-267	MHOM/SD/1997/LEM3472^a^	DQ449694.1	A	**.**	C	A
MON-309	ITOB/TR/2005/CUK2	EU545241.1	**.**	**.**	C	**.**
MON-309	MHOM/TR/2005/CUK1	EU545240.1	**.**	**.**	C	**.**
ni	ITOB/TR/2007/CUK10	EU545242.1	**.**	**.**	C	**.**
non-MON-1	MHOM/TR/2000/OG-VL	EU545243.1	**.**	**.**	**.**	**.**
ni	ni	DQ449698.1	**.**	**.**	**.**	**.**

Zymodeme sequences obtained in this study are in bold

*Abbreviation*: *ni* not indicated

*Key*: **.** consensus sequence, – sequence not available

^a^Initially assigned to *L. infantum* and successively designated *L. donovani* [18]

**Table 6:** Polymorphisms in glucose-6-phosphate isomerase (*gpi*)

Zymodeme	Strain/isolate	GenBank ID	703	1503	1807	1831
MON-1	MHOM/FR/1978/LEM75	AJ620617.1	T	G	C	G
MON-1	MHOM/TN/1980/IPT1	AJ620647.1	**.**	**.**	**.**	A
MON-1	MHOM/PT/2000/IMT260	AM157725.1	**.**	**.**	**.**	**.**
MON-1	MHOM/ES/1986/BCN16	AM157724.1	**.**	**.**	**.**	**.**
MON-1	MHOM/FR/1997/LSL29	AM157723.1	**.**	**.**	**.**	**.**
MON-1	MHOM/ES/1993/PM1	AM157722.1	**.**	**.**	**.**	**.**
MON-1	MHOM/FR/1995/LPN114	AM157721.1	**.**	**.**	**.**	**.**
**MON-1**	**MHOM/TN/1980/IPT1**	**MF288905**	–	**.**	–	–
**MON-1**	**MHOM/FR/78/LEM75**	**MF288906**	–	**.**	–	–
**MON-1**	**Isolate 31u**	**MF288907**	–	**.**	–	–
**MON-1**	**Isolate 49u**	**MF288908**	–	**.**	–	–
**MON-1**	**Isolate 791**	**MF288909**	–	**.**	–	–
**MON-1**	**Isolate 10816**	**MF288910**	–	**.**	–	–
**MON-1**	**Isolate V2921**	**MF288911**	–	**.**	–	–
**MON-24**	**MHOM/DZ/82/LIPA59**	**MF288912**	–	**.**	–	–
MON-27	MHOM/IT/1979/Francesca	AM117192.1	**.**	**.**	**.**	R
**MON-29**	**MHOM/ES/82/BCN1**	**MF288913**	–	**.**	–	–
MON-29	MHOM/FR/1996/LEM3249	AJ620618.1	**.**	**.**	**.**	**.**
MON-34	MHOM/CN/1980/A	AJ620637.1	**.**	**.**	**.**	A
**MON-72**	**MHOM/IT/86/ISS218**	**MF288914**	–	**.**	–	–
MON-78	MHOM/MT/1985/BUCK	AJ620619.1	**.**	**.**	**.**	A
MON-81	MHOM/SD/1962/3S^a^	AJ620629.1	C	**.**	T	**.**
MON-183	MHOM/ES/1991/LEM2298	AJ620620.1	**.**	T	**.**	**.**
MON-198	MHOM/ES/1988/LLM175	AJ620630.1	**.**	T	**.**	**.**
MON-199	MHOM/ES/1992/LLM373	AJ620631.1	**.**	T	**.**	R
**MON-201**	**MHOM/IT/93/ISS822**	**MF288915**	–	**.**	–	–
MON-228	MHOM/IT/1994/ISS1036	AJ620632.1	**.**	K	**.**	**.**
MON-267	MHOM/SD/1997/LEM3472^a^	AJ620634.1	C	**.**	T	**.**

Zymodeme sequences obtained in this study are in bold

*Key*: **.** consensus sequence, – sequence not available

^a^Initially assigned to *L. infantum* and successively designated *L. donovani* [18]

**Table 7:** Polymorphisms in glucose-6-phosphate dehydrogenase (*g6pdh*)

Zymodeme	Strain/isolate	GenBank ID	45	315	348	359	403	468	690	1191
MON-1	MHOM/FR/78/LEM75	DQ449770.1	T	C	T	A	G	T	C	C
MON-1	MHOM/PT/2000/IMT260	DQ449775.1	**.**	**.**	**.**	**.**	**.**	**.**	**.**	**.**
MON-1	MHOM/ES/1986/BCN16	DQ449774.1	**.**	**.**	**.**	**.**	**.**	**.**	**.**	**.**
MON-1	MHOM/FR/1997/LSL29	DQ449773.1	**.**	**.**	**.**	W	**.**	**.**	**.**	**.**
MON-1	MHOM/ES/1993/PM1	DQ449772.1	**.**	**.**	**.**	**.**	**.**	**.**	**.**	**.**
MON-1	MHOM/FR/1995/LPN114	DQ449771.1	**.**	**.**	**.**	**.**	**.**	**.**	**.**	**.**
MON-1	MHOM/BL/67/ITMAP263	KU175220.1	–	**.**	**.**	**.**	**.**	**.**	**.**	**.**
**MON-1**	**MHOM/TN/80/IPT1**	**MF479731**	**.**	**.**	**.**	**.**	**.**	**.**	**.**	–
**MON-1**	**MHOM/FR/78/LEM75**	**MF479732**	**.**	–	–	–	–	**.**	**.**	–
**MON-1**	**Isolate 49u**	**MF479733**	**.**	**.**	**.**	**.**	**.**	**.**	**.**	–
**MON-1**	**Isolate 31U**	**MF479734**	**.**	**.**	**.**	–	–	–	**.**	–
**MON-1**	**Isolate 791**	**MF479735**	**.**	**.**	**.**	**.**	**.**	**.**	**.**	–
**MON-1**	**Isolate 10816**	**MF479736**	**.**	**.**	**.**	**.**	**.**	**.**	**.**	–
**MON-1**	**Isolate V2921**	**MF479737**	**.**	**.**	**.**	**.**	**.**	**.**	**.**	–
MON-11	MHOM/FR/1980/LEM189	DQ449783.1	C	**.**	**.**	T	**.**	**.**	**.**	**.**
**MON-24**	**MHOM/DZ/82/LIPA59**	**MF479738**	C	**.**	**.**	**.**	**.**	**.**	**.**	–
**MON-29**	**MHOM/ES/82/BCN1**	**MF479739**	C	**.**	**.**	T	**.**	**.**	**.**	–
MON-29	MHOM/FR/1996/LEM3249	DQ449776.1	C	**.**	**.**	W	**.**	**.**	**.**	–
**MON-72**	**MHOM/IT/86/ISS218**	**MF479740**	**.**	**.**	**.**	**.**	**.**	**.**	**.**	–
MON-77	MCAM/ES/86/LEM935	DQ449797.1	**.**	**.**	**.**	W	**.**	**.**	**.**	–
MON-78	MHOM/MT/1985/BUCK	DQ449784.1	C	**.**	**.**	**.**	**.**	**.**	**.**	**.**
MON-81	MHOM/SD/1962/3S^a^	DQ449787.1	C	G	C	**.**	**.**	**.**	T	T
MON 105	MHOM/ES/2001/LLM1026	DQ449799.1	**.**	**.**	**.**	**.**	**.**	**.**	**.**	**.**
MON-183	MHOM/ES/1991/LEM2298	DQ449777.1	**.**	**.**	**.**	W	**.**	**.**	**.**	**.**
MON-188	MHOM/IT/1993/ISS800	DQ449791.1	C	**.**	**.**	**.**	**.**	**.**	**.**	**.**
MON-198	MHOM/ES/1988/LLM175	DQ449788.1	C	**.**	**.**	T	**.**	**.**	**.**	**.**
MON-199	MHOM/ES/1992/LLM373	DQ449789.1	**.**	**.**	**.**	W	**.**	**.**	**.**	**.**
**MON-201**	**MHOM/IT/93/ISS822**	**MF479741**	**.**	**.**	**.**	**.**	**.**	**.**	**.**	**.**
MON-228	MHOM/IT/1994/ISS1036	DQ449790.1	**.**	**.**	**.**	**.**	**.**	**.**	**.**	**.**
MON-253	MHOM/ES/1996/LLM580	DQ449798.1	**.**	**.**	**.**	**.**	A	**.**	**.**	**.**
MON-267	MHOM/SD/1997/LEM3472^a^	DQ449792.1	C	G	C	**.**	**.**	**.**	T	T
MON-267	MCAN/SD/2000/LEM3988	KU175214.1	–	G	C	**.**	**.**	**.**	T	**.**
MON-309	ITOB/TR/2005/CUK2	EU545237.1	C	**.**	**.**	**.**	**.**	**.**	**.**	**.**
MON-309	MHOM/TR/2005/CUK1	EU545236.1	C	**.**	**.**	**.**	**.**	**.**	**.**	**.**
ni	ITOB/TR/2007/CUK10	EU545238.1	C	**.**	**.**	**.**	**.**	**.**	**.**	**.**
ni	MHOM/CN/93/KXG-XU	JX021334.1	–	**.**	C	**.**	**.**	C	**.**	**.**
ni	MHOM/CN/94/KXG-LIU	JX021333.1	–	**.**	C	**.**	**.**	C	**.**	**.**
non-MON-1	MHOM/TR/2000/OG-VL	EU545239.1	**.**	**.**	**.**	**.**	**.**	**.**	**.**	**.**
ni	Isolate RRR-B	DQ449796.1	**.**	**.**	**.**	**.**	**.**	**.**	**.**	**.**
ni	MHOM/PA/78/WR285	KU175216.1	–	**.**	**.**	**.**	**.**	**.**	**.**	**.**
ni	Isolate D38	KU175221.1	–	**.**	**.**	**.**	**.**	**.**	**.**	**.**
ni	Isolate D33	KU175219.1	–	**.**	**.**	**.**	**.**	**.**	**.**	**.**
ni	Isolate D36D	KU175218.1	–	**.**	**.**	**.**	**.**	**.**	**.**	**.**
ni	Isolate E9D	KU175217.1	–	**.**	**.**	**.**	**.**	**.**	**.**	**.**
ni	JPCM5	XM_001468358.1	**.**	**.**	**.**	**.**	**.**	**.**	**.**	**.**

Zymodeme sequences obtained in this study are in bold

*Abbreviation*: *ni* not indicated

*Key*: **.** consensus sequence, – sequence not available

^a^Initially assigned to *L. infantum* and successively designated *L. donovani* [18]

**Table 8:** Polymorphisms in mannose phosphate isomerase (*mpi*)

Zymodeme	Strain/isolate	GenBank ID	300	392	486	637	798
MON-1	MHOM/FR/1978/LEM75	DQ449737.1	C	A	G	A	C
MON-1	MHOM/FR/1995/LPN114	DQ449738.1	**.**	**.**	**.**	**.**	**.**
MON-1	MHOM/ES/1993/PM1	DQ449739.1	**.**	**.**	**.**	**.**	**.**
MON-1	MHOM/FR/1997/LSL29	DQ449740.1	**.**	**.**	**.**	**.**	**.**
MON-1	MHOM/ES/1986/BCN16	DQ449741.1	**.**	**.**	**.**	**.**	**.**
MON-1	MHOM/PT/2000/IMT260	DQ449742.1	**.**	**.**	**.**	**.**	**.**
**MON-1**	**MHOM/TN/1980/IPT1**	**MF462101**	**.**	**.**	**.**	**.**	**.**
**MON-1**	**MHOM/FR/78/LEM75**	**MF462102**	**.**	–	–	**.**	**.**
**MON-1**	**Isolate 31u**	**MF462103**	**.**	**.**	**.**	**.**	**.**
**MON-1**	**Isolate 791**	**MF462104**	**.**	**.**	**.**	**.**	**.**
**MON-1**	**Isolate 10816**	**MF462105**	**.**	**.**	–	**.**	**.**
**MON-1**	**Isolate V2921**	**MF462106**	**.**	**.**	**.**	**.**	**.**
**MON-1**	**Isolate 49u**	**MF462107**	**.**	**.**	**.**	**.**	**.**
MON-11	MHOM/FR/1980/LEM189	DQ449750.1	**.**	**.**	**.**	**.**	**.**
**MON-24**	**MHOM/DZ/82/LIPA59**	**MF462108**	**.**	**.**	**.**	**.**	**.**
**MON-29**	**MHOM/ES/81/BCN1**	**MF462109**	**.**	**.**	**.**	**.**	**.**
MON-29	MHOM/FR/1996/LEM3249	DQ449743.1	**.**	**.**	**.**	**.**	**.**
**MON-72**	**MHOM/IT/86/ISS218**	**MF462110**	**.**	**.**	**.**	**.**	**.**
MON-78	MHOM/MT/1985/BUCK	DQ449751.1	**.**	**.**	**.**	**.**	**.**
MON-81	MHOM/SD/1962/3S^a^	DQ449754.1	**.**	**.**	**.**	**.**	**.**
MON-136	MHOM/IT/1990/ISS510	DQ449766.1	**.**	**.**	A	R	**.**
MON-183	MHOM/ES/1991/LEM2298	DQ449744.1	**.**	**.**	**.**	**.**	**.**
MON-188	MHOM/IT/1993/ISS800	DQ449758.1	**.**	**.**	**.**	G	**.**
MON-198	MHOM/ES/1988/LLM175	DQ449755.1	**.**	**.**	**.**	**.**	**.**
MON-199	MHOM/ES/1992/LLM373	DQ449756.1	**.**	**.**	**.**	**.**	**.**
**MON-201**	**MHOM/IT/93/ISS822**	**MF462111**	**.**	**.**	**.**	R	**.**
MON-228	MHOM/IT/1994/ISS1036	DQ449757.1	**.**	**.**	**.**	**.**	**.**
MON-267	MHOM/SD/1997/LEM3472^a^	DQ449759.1	T	R	**.**	**.**	T
non-MON-1	MHOM/TR/2000/OG-VL	EU545251.1	**.**	**.**	**.**	**.**	**.**
ni	MHOM/TR/2005/CUK1	EU545248.1	**.**	**.**	**.**	**.**	**.**
ni	ITOB/TR/2005/CUK2	EU545249.1	**.**	**.**	**.**	**.**	**.**
ni	ITOB/TR/2007/CUK10	EU545250.1	**.**	**.**	**.**	**.**	**.**
ni	MHOM/CN/94/KXG-LIU	JX021373.1	T	**.**	**.**	**.**	T
ni	MHOM/CN/93/KXG-XU	JX021374.1	T	**.**	**.**	**.**	T
ni	Isolate RRR-B	DQ449763.1	Y	**.**	**.**	**.**	Y

Zymodeme sequences obtained in this study are in bold

*Abbreviation*: *ni* not indicated

*Key*: **.** consensus sequence, – sequence not available

^a^Initially assigned to *L. infantum* and successively designated *L. donovani* [18]

**Fig. 1:**
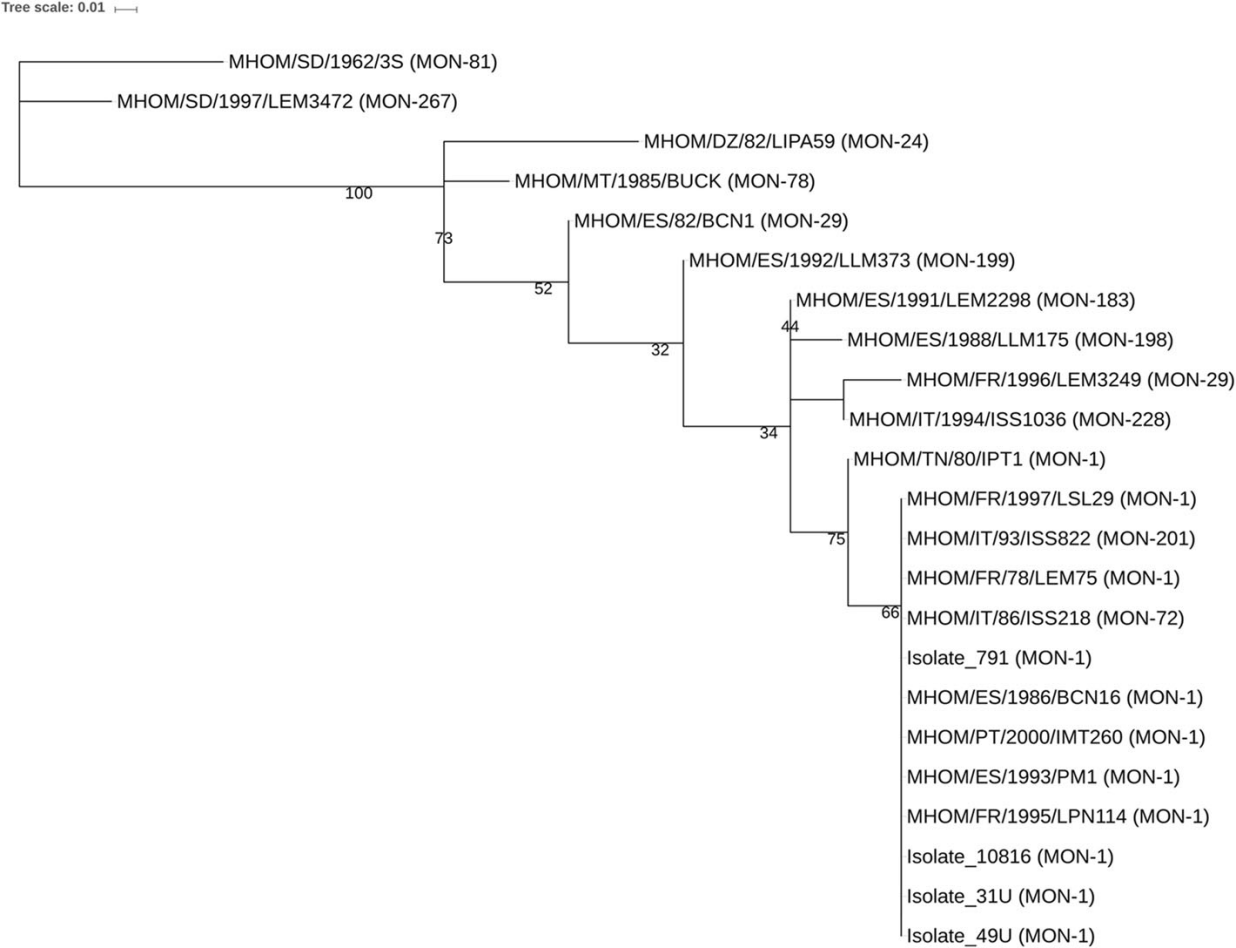
Phylogenetic tree of 23 strains based on the multilocus sequence data. The sequencing data for six genes (*me*, *pgd*, *icd*, *gpi*, *g6pdh*, *mpi*) were used to build a 3578 bp long sequence. The tree was constructed using the Maximum Likelihood method based on the GTR+I model using PhyML 3.0 software. Bootstrap values were calculated from 100 replications. Numbers below branches represent bootstrap support

The concatenated partial sequences for *me*, *pgd*, *icd*, *gpi*, *g6pdh*, *mpi* from 23 strains were used for phylogenetic tree construction (Fig. 1, Additional file 2: Table S1). The tree showed a 100% bootstrap support for the *L. infantum* strains and a 75% support for the clade including all strains of MON-1, 72, 201 zymodemes. Within this clade, the MHOM/TN/80/IPT1 strain differed from the bulk of closely related strains (bootstrap support value of 66%) (Fig. 1). This made it possible to identify the most common genotypes in Europe using a simple screening method (see below).

### Exploiting sequence polymorphisms for typing

**Fig. 2:**
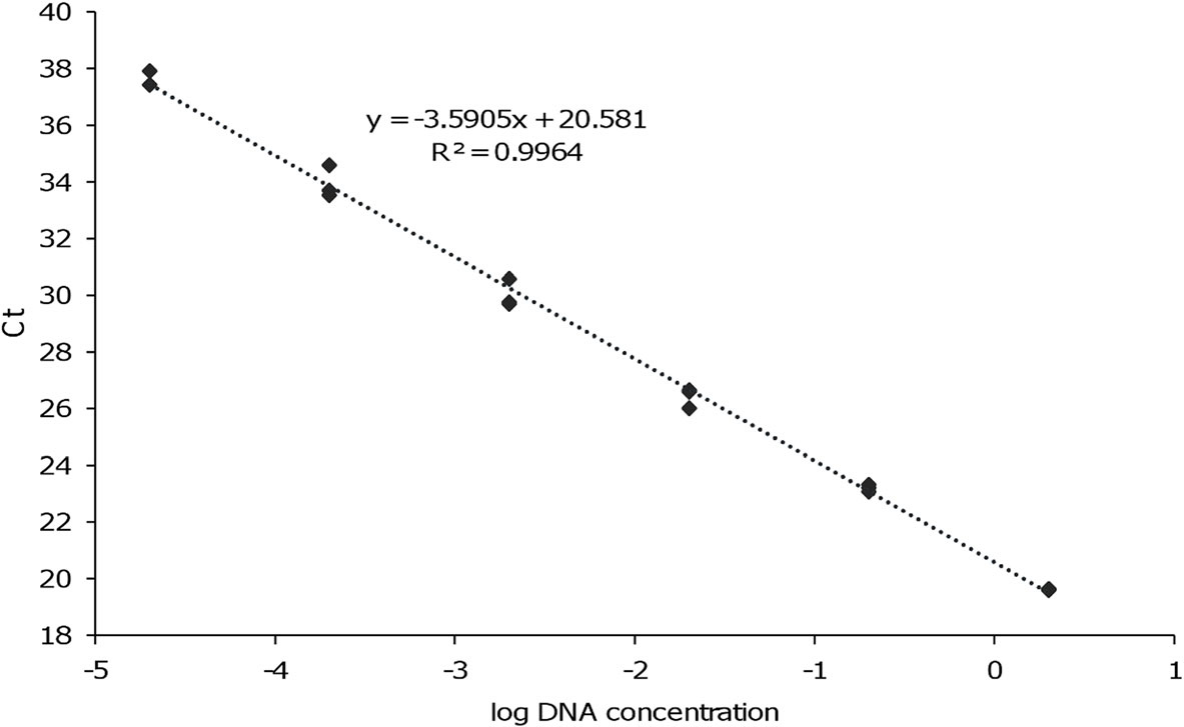
Linear correlation between the log of DNA concentration and Ct values. The qPCR-MEint curve was obtained amplifying serial dilutions of genomic DNA (from 2 to 2 × 10^-5^ ng/reaction tube) purified from *L. infantum* MHOM/FR/78/LEM75 (y = -3.59x + 20.58). Each reaction was performed in triplicate. Reaction efficiency = 90%, *R*^2^ = 0.996

As shown in Fig. 1, the clade including zymodemes MON-1, 72, 201 could be discriminated from all other zymodemes with the exception of the MHOM/TN/80/IPT1 strain. The sequence comparison analysis showed that this group could be discriminated by exploiting the polymorphism 390T>G in the *me* gene sequence (Table 3). In fact, MON-1, 72, 201 present a T in position 390 (genotype 390T), while the other zymodemes present a G (genotype 390G). Due to this observation, we developed a HRM-based assay to monitor the SNP at position 390. To this end, two internal primers were designed upstream and downstream nucleotide 390, and used for a new qPCR assay (qPCR-MEint) (Additional file 1: Figure S1a). The qPCR-MEint showed a linear correlation between the log of DNA concentration (from 2 to 2 × 10^-5^ ng/reaction) and Ct values, with a reaction efficiency of 90% (Fig. 2).

**Fig. 3:**
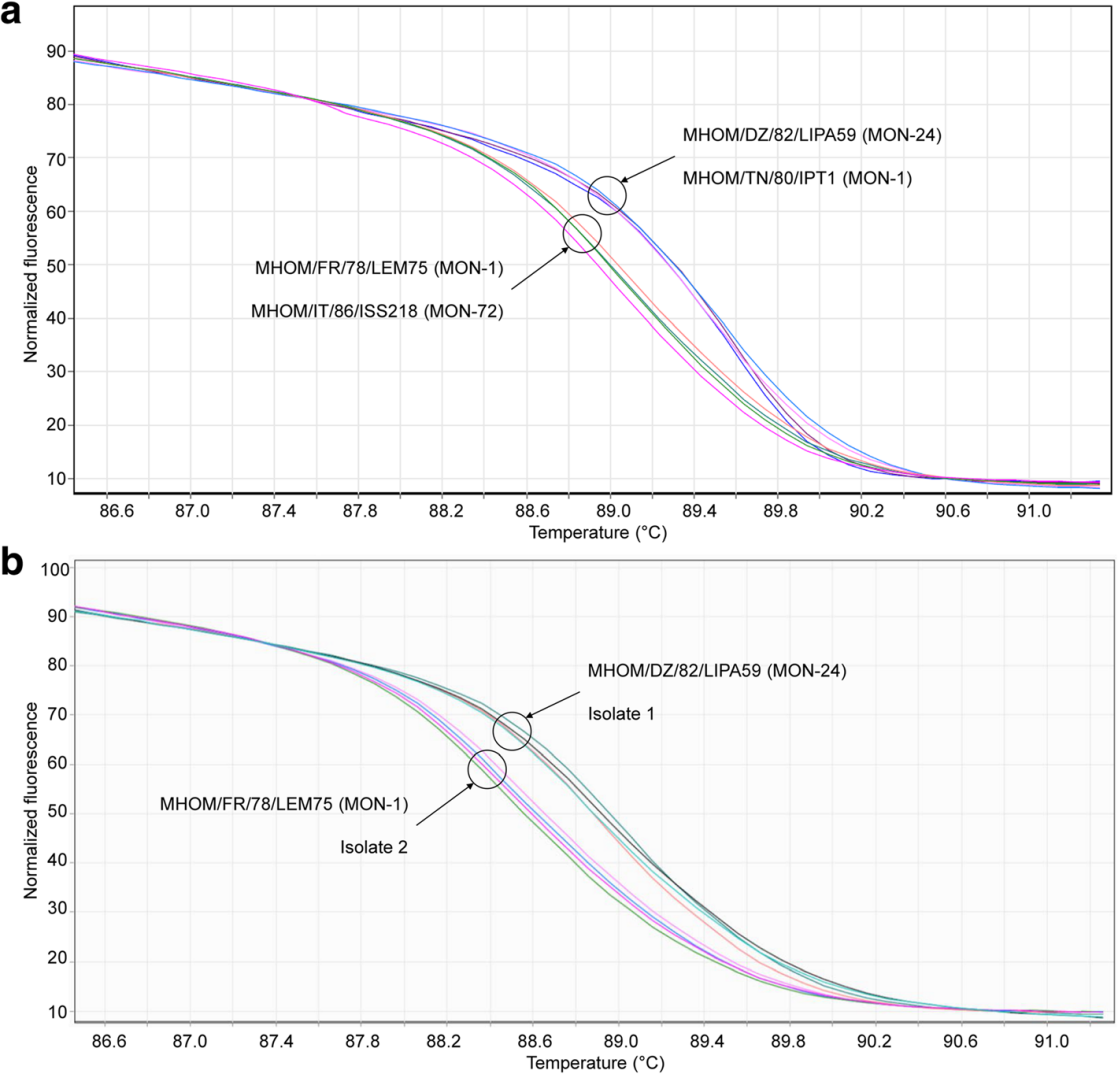
HRM analysis of amplicons with genotypes 390G and 390T. **a** HRM profiles obtained from qPCR-MEint amplicons of strains MHOM/TN/80/IPT1 and MHOM/DZ/82/LIPA59 (genotype 390G) and amplicons of strains MHOM/FR/78/LEM75 and MHOM/IT/86/ISS218 (genotype 390T). **b** The analysis of HRM profiles allowed the assignation of amplicons of clinical isolates 1 and 2 to genotypes 390G and 390T, respectively, with a confidence > 95% in at least one replicate. Strains MHOM/DZ/82/LIPA59 (genotype 390G) and MHOM/FR/78/LEM75 (genotype 390T) were used as reference. Analyses were performed in duplicates

HRM analysis allowed us to distinguish amplicons with genotype 390G (i.e. MHOM/TN/80/IPT1 and MHOM/DZ/82/LIPA59 strains) from amplicons with genotype 390T (i.e. MHOM/FR/78/LEM75 and MHOM/IT/86/ISS218 strains) (Fig. 3a). Furthermore, using the PCR product from strains MHOM/DZ/82/LIPA59 and MHOM/FR/78/LEM75 as reference, two *L. infantum* clinical isolates (isolate 1 and 2) [32] were typed with qPCR-MEint followed by HRM analysis. The HRM analysis tool of Rotor-Gene 6000 software allowed for the assignment of amplicons of clinical isolate 1 and 2 to genotype 390G and genotype 390T, respectively, with a confidence > 95% in at least one replicate (Fig. 3b). These results have been confirmed by PCR product sequencing (Additional file 3: Figure S2).

### Application of HRM analysis in clinical samples

To test the feasibility of the HRM-based typing approach without parasite cultivation, the qPCR-MEint followed by HRM analysis was performed using DNA extracted from canine clinical samples [31, 33] and a human clinical sample (blood), using PCR product from strains MHOM/DZ/82/LIPA59 and MHOM/FR/78/LEM75 as reference. The human peripheral venous blood sample was obtained during routine diagnosis of a patient with VL by venipuncture of the upper limb. The HRM analysis allowed for the assignment of all samples to group 390T (Table 9). The results were confirmed by PCR product sequencing (Additional file 4: Figure S3).

**Table 9:** HRM analysis results of clinical samples

Name	Genotype	Confidence %
MHOM/FR/78/LEM75	390T	98.02
MHOM/FR/78/LEM75	390T	98.02
MHOM/DZ/82/LIPA59	390G	99.90
MHOM/DZ/82/LIPA59	390G	99.90
62 bc	390T	85.05
62 bc	390T	82.99
77 sx	390T	79.40
77 sx	390T	94.41
vea bm	390T	97.69
vea bm	390T	97.38
Psalb	390T	86.23
Psalb	390T	77.97

*Abbreviations*: 62 bc, canine sample, buffy coat; 77 sx, canine sample, conjunctival swab; vea bm, canine sample, bone marrow; psalb, human sample, blood

## Discussion

Epidemiological studies examining the dynamic balance between host, vector, and pathogen populations is an important aspect of *Leishmania* infections, and the importance of reservoirs alternative to the canine population (e.g. lagomorphs, wolves) is currently being investigated [34, 35]. For example, in Italy, *L. infantum* zymodemes retrieved in infected dogs are primarily represented by MON-1 and MON-72, while infection in humans is caused by a more heterogeneous zymodeme population, suggesting that the canine population is not the only reservoir for all *L. infantum* zymodemes [15].

In this study, we investigated the genetic diversity within seven *L. infantum* genes, aiming to provide information that could aid in the development of tools for fast genetic characterization and epidemiological screening of *L. infantum* in mammalian host and/or vectors. In particular, we investigated whether it was possible to distinguish the genotype associated with the most common zymodemes in the Mediterranean area (i.e. MON-1 and MON-72), from the genotype associated with all other zymodemes, therefore limiting the number of samples needing costly and time-consuming characterizations. To this end, we collected and aligned all *L. infantum* gene sequences of seven enzymes (*me*, *pgd*, *icd*, *gpi*, *g6pdh*, *mpi*, *pgm*) available in GenBank to identify the regions that were more polymorphic. These regions were amplified in 11 other *L. infantum* strains available in our laboratory to increase the number of sequences to compare. The phylogenetic analysis of the concatenated sequences showed that the group including zymodemes MON-1, 72, 201 clustered separately. Zymodeme MON-201 has been found only in some HIV-positive individuals [12, 36] and in one case of feline leishmaniasis [37]; therefore, its retrieval may be considered rare. Notably, the genetic distance of MON-81 and MON-267 from other *L. infantum* zymodemes was confirmed (Fig. 1, Additional file 2: Table S1). In fact, MON-81 and MON-267, initially considered *L. infantum*, have been successively assigned to *L. donovani* [18].

Remarkably, the silent polymorphism at position 390 (T/G) of the *me* gene distinguishes strains of MON-1, 72, 201 (genotype 390T) from strains of other zymodemes (genotype 390G) (Table 3). This could be important for epidemiological applications, as MON-1 is the most common zymodeme of *L. infantum* in Europe. The only exception was the reference strain MHOM/TN/80/IPT1, which presents a guanine at nucleotide 390. This may be due to the geographical origin of this strain (Tunisia) compared to the European strains/isolates. It is also noteworthy that, among the MON-1 strains, only strain MHOM/TN/80/IPT1 cannot be distinguished from the MON-98 strains (considering that only *me* sequences were available for this MON) (Table 3). This could be in agreement with results reported by Haralambous et al. [24], which showed that some MON-1 and MON-98 zymodemes were not distinguished with a PCR-based typing method using the K26 antigen-coding gene as target sequence. Notably, the use of a real-time PCR-based assay with internal primers followed by HRM analysis allowed for the quick differentiation of sequences with the polymorphysm 390T>G not only in *L. infantum* isolates/strains, but also in clinical samples, enabling the potential application of this approach in population screening without parasite isolation and culturing.

Many marker sequences can be taken into consideration for genotyping purposes. The most suitable marker sequence depends on the level of resolution needed: highly variable markers can distinguish closely related genotypes but may not faithfully represent more distant relationships, while moderately variable markers will not be able to evidence the differences between closely related samples. The assay that we propose would not be able to distinguish closely related genotypes; therefore, it could be used to differentiate between relapse and re-infection only in cases where different 390T/G genotypes were found. However, it could be useful for epidemiological screening in regions where MLEE data are available, to rapidly identify zymodemes different from MON-1, 72 or 201 (e.g. for rapid screening of *L. infantum* genotypes in mammalian hosts). Once attributed a genotype (390T or 390G), other tests (e.g. MLEE or MLST) can be performed, depending on the level of resolution needed and on the classification system with which the results are to be compared.

Other zymodemes (i.e. MON-24, MON-78 and MON-199) could be identified by exploiting the unique SNP found across the seven gene sequences. In particular MON-24, which is another common zymodeme [12, 38], could be differentiated by exploiting the polymorphisms 204T>C in the *icd* gene (Table 5) by using a HRM-based assay similar to the assay described here.

A possible limitation of this study is the unavailability of some zymodeme sequences in the database. Moreover, in many cases, only one strain with sequence information per zymodeme was available. This is important to keep in mind, since strains of the same zymodeme could present different genotypes (e.g. MON-29 in *me*, *icd* and *g6pdh* genes) (Additional file 2: Table S1).

## Conclusions

A total of 77 new sequences of seven genes encoding for metabolic enzymes in *L. infantum* isolates/strains have been produced and deposited in the GenBank database. The analysis of these sequences, together with sequences available in the database, allowed for the identification of genetic polymorphisms exploitable to differentiate the most common *L. infantum* zymodemes in the Mediterranean basin. In particular, a HRM-based assay aimed to differentiate the genotype 390T and 390G in the *me* gene was developed. The genotype 390T correlated with zymodemes MON-1, 72, 201, allowing for the rapid identification of the majority of *L. infantum* genotypes. Once a parasite is attributed to genotype 390T or 390G, other tests (e.g. MLEE, MLST or MLMT) can be performed if more detailed information is needed. This assay has been successfully applied to clinical samples, demonstrating its potential applicability in investigating the role of other mammalian hosts in epidemiological screening.

## Additional files

Additional file 1: **Figure S1.** Reference sequences and position of primers used in this study. **a** *L. infantum* strain MHOM/FR/1978/LEM75 cytosolic NADP-malic enzyme (me) gene, partial cds. (GenBank: DQ449701.1). Primers amplifying 5' region and 3' region are boxed and underlined, respectively. Internal primers are in bold. **b** *L. infantum* gene for phosphogluconate dehydrogenase (decarboxylating), strain MHOM/FR/1978/LEM75 (GenBank: AM157139.1). **c** *L. infantum* strain MHOM/FR/1978/LEM75 mitochondrial isocitrate dehydrogenase (icd) gene, complete cds (GenBank: DQ449672.1). **d** *L. infantum* gpi gene for glucose-6-phosphate isomerase, strain MHOM/FR/1978/LEM75 (GenBank: AJ620617.1). **e** *L. infantum* strain MHOM/FR/1978/LEM75 glucose-6-phosphate dehydrogenase (g6pdh) gene, complete cds (GenBank:DQ449770.1). **f** *L. infantum* strain MHOM/FR/1978/LEM75 mannose phosphate isomerase (mpi) gene, complete cds (GenBank: DQ449737.1). **g** *L. infantum* isolate MCAN/AR/10/MDP1 phosphoglucomutase gene, partial cds (GenBank: KJ643214.1). (DOCX 20 kb)

Additional file 2: **Table S1.** Summary of allelic profile and genotype of *L. infantum* zymodemes considered in this study. (DOCX 18 kb)

Additional file 3: **Figure S2.** Partial sequences of qPCR-MEint amplification products from reference strains and clinical isolates. Electropherograms encompassing polymorphic nucleotide at position 390 (arrows) are represented. (PDF 72 kb)

Additional file 4: **Figure S3.** Partial sequences of qPCR-MEint amplification products from clinical samples. Electropherograms encompassing polymorphic nucleotide at position 390 (arrows) are represented. (PDF 570 kb)

## Abbreviations

qPCR: Quantitative real-time PCR
VL: Visceral leishmaniasis
CL: Cutaneous leishmaniasis
HRM: High-resolution melting
MLEE: Multilocus enzyme electrophoresis
MLST: Multilocus sequence typing
MLMT: Multilocus microsatellite typing
