# Supplementary material for: Exploiting genetic polymorphisms in metabolic enzymes for rapid screening of Leishmania infantum genotypes
Source: Parasit Vectors. 2018 Nov 1;11:572. doi: 10.1186/s13071-018-3143-7 (PMC6211443; doi:10.1186/s13071-018-3143-7)
Supplement: Supplementary file 1 — Figure S1. Reference sequences and position of primers used in this study. a L. infantum strain MHOM/FR/1978/LEM75 cytosolic NADP-malic enzyme (me) gene, partial cds. (GenBank: DQ449701.1). Primers amplifying 5' region and 3' region are boxed and underlined, respectively. Internal primers are in bold. b L. infantum gene for phosphogluconate dehydrogenase (decarboxylating), strain MHOM/FR/1978/LEM75 (GenBank: AM157139.1). c L. infantum strain MHOM/FR/1978/LEM75 mitochondrial isocitrate dehydrogenase (icd) gene, complete cds (GenBank: DQ449672.1). d L. infantum gpi gene for glucose-6-phosphate isomerase, strain MHOM/FR/1978/LEM75 (GenBank: AJ620617.1). e L. infantum strain MHOM/FR/1978/LEM75 glucose-6-phosphate dehydrogenase (g6pdh) gene, complete cds (GenBank:DQ449770.1). f L. infantum strain MHOM/FR/1978/LEM75 mannose phosphate isomerase (mpi) gene, complete cds (GenBank: DQ449737.1). g L. infantum isolate MCAN/AR/10/MDP1 phosphoglucomutase gene, partial cds (GenBank: KJ643214.1). (DOCX 20 kb) [file 13071_2018_3143_MOESM1_ESM.docx]

**a**

1 CGCAACCGCT TCACCAATAA GGGCACAGCC TTTACCGCAG CAGAGCGGTC GCACATGAAC

61 GTGGAAGGGT TGCTGCCGCC CTCTGTCGAG ACCCTCGATG ATCAGGTGGA GCGGTACTGG

121 GATCAGCTGA ACCGTTTCAA CGAGCCGATC AACCGCTATC AGTTGCTGCG CAACGTGCAG

181 AACACGAACG TCACCCTCTA CTACGCCATC TTGACGCGGT ACCTGAAGCA GACACTGCCG

241 ATCGTGTACA CACCGACCGT CGGCGAGGCC TGCCAGCGCT ACGGTGACCT CTATCAGAAG

301 GACCACGGAC TGTACCTCGA CGTCGCCATC AAGGGCAAGG TGAGGAAGCT **GATTCAGAAC**

361 **CTTCGCAAGA CGA**ACGTCGA CGTCATCGTT ATCACCGATG GCTCCCGCAT TCTCGGCCTG

421 GGCGACCTCG GCGCCAACGG CATCG**GCATC AGCATCGGCA AGTG**CTCCCT GTACGTCGCT

481 GCGGGCGGTG TGAAGCCGAG CCGCGTTCTG CCGGTCGTCA TGGACGTTGG CACAAACAAC

541 CTCGAGCTCC GCAACAACCC GCTTTATCTC GGTTTGCGCA AGCCGCGGTG CGGCGACGCC

601 GACTTTTACG CTCTGCTGGA CGAGTTCATG GAAGCTGTGA AGGACACCTG GCCCTCCGCT

661 GTCGTGCAGT TCGAGGACTT CAGCAACAAC CACTGCTTCG ACATGCTGGA GCGCTACCAA

721 AAGAAGTACC GCTGCTTCAA CGACGATATC CAGGGCACCG GCGCCGTCAT AGCTGCTGGT

781 TTCCACACGG CGGTGAAGCT AAGCAAGATC CCGATGGAGC AGCAGCGCAT CGTCTTCTTC

841 GGCGCCGGCT CTGCCGCGAC CGGTGTGGCG GAGAGCATCG CCGACCTCGC CGCTGAGGCC

901 GGGATGAAGA AAGAGGACGT CAAGAAGAGC ATCTTCTTCG TCGACTCGAT GGGCATGGTG

961 GCCACCAACC GCGGTGACAA GCTGGCCAAG CACAAGCTGG GATGGGCCCG CACCGACATC

1021 CCTGACGCAG TTATTGCAAG CCTGAAGACT CTCGAGGACG TTGTGCGCTA CGTGCGGCCG

1081 ACCGCGCTCA TCGGCCTCGG CGCCACCGCC AACGTCTTTT CGCGCGAGAT TGTGGAGTTT

1141 CTGCACTCGT GCTGCCCTCA CCCGATCATA TTCCCGCTGT CAAATCCGTC CAGCAAAGCC

1201 GAAATTGTGC CGGCGAACGC ATACAAGTGG ACGAACGGCG ATGCCATCGT GGCCTCCGGC

1261 AGCCCCTTTC CTGAGACGGT CGTCAGCGGC CGCACGCTGC AACCATCGCA AGGCAACAAC

1321 CTGTACATCT TCCCCGGGGT GGGTCTCGGC TGCTGCATTG CTCAGCCGCC GTACATCCCG

1381 CAGGAGGTGC TGGTGGCGGC CGCTGCCTGC CTGAGCACGC TGGCCACGCC GGACGACCTC

1441 GCCAAGGGAC AGCTGTACCC GTCTATTGAG GAGGTGCGCC GCGTGTCGCG TGAGGTGGCC

1501 GTGGCGTGTA TTCAGAAGCT ACAGGAGCTG GGGCTGGCCA AGGCAGATCT GCCAGATAAC

1561 CGCCCAGACC TGATGAAGCT GGTGAAGACG GCTTTCTGGG AGCCGCGCTA CTTGCCCGAG

1621 AACTACTACC TGGAGAAGGA GTTG

**b**

1 ATGTCGAACG ACCTCGGTAT TATCGGCCTC GGTGTCATGG GCGCGAATCT CGCCCTGAAC

61 ATCGCCGAGA AGGGATTCAA AGTTGCCGTC TTCAACCGCA CCTCCGCGAA GACGGCGTCG

121 TTTCTCAAGG AGCATGAGAG CGAGAAATTT GCCGCCAACC TGAATGGCTA CGAGACCATG

181 AAGGCGTTCG CTGCGTCCCT CAAGAAGCCG CGCCGCGCGT TCATTCTCGT CCAGGCCGGC

241 GCCGCTACGG ACTCTACGAT CGAGCAGCTC AAGGAAGTGC TCGAGAATGG CGACATCATA

301 ATTGACACTG GCAATGCGAA CTTCAAGGAC CAGGACAAGC GTGCCGCGCA GTTGGAGAGC

361 CAGGGTCTCC GCTTCCTCGG CATGGGCATC TCCGGTGGTG AGGAGGGTGC GCGCAAGGGG

421 CCGGCCTTCT TCCCGGGTGG CACACCAAGC GTGTGGGAGG AGGTGCGCCC GATCGTGGAG

481 GCGGCTGCGG CCAAGGCTGA GGACGGTCGC CCGTGCGTGA CTTTCAACGG CAAGGGCGGC

541 GCTGGATCCT GCGTGAAGAT GTATCACAAC GCTGGTGAGT ACGCCGTGCT GCAGATCTGG

601 GGTGAGGCGT ACACCGCCCT GCTTGCCTTC GGCTTCGACA ACGATCAGAT CGCCGACGTG

661 TTCGAGTCGT GGAAGGCAGA TGGCTTCCTC AAATCCTACA TGCTCGACAT TTCCATTGCC

721 GCTTGCCGCG CGAGAGAGGC AGCAGGCAAC TATCTGTCAG AGAAGGTCAA GGACCGCATC

781 GGCTCCAAGG GCACTGGCCT GTGGTCTGCC CAGGAGGCTC TGGAGGTTGG CGTGCCAGCG

841 CCCTCGCTCA GCATGGCCGT CATCTCACGC CAGATGACCA TGTGCAAAGA GGAGCGTATT

901 GCGAACTGCA AGGCATTCCC CAACTTCCCT CGTGGCCCGT CTGCGGAAGC CAGGGACAAG

961 TCCCCGAACT CCCCCGACGC GAAGCAGCTG TACCACGCCG TCAGCCTCTG CATCATCGCG

1021 AGCTACGCGC AGATGTTCCA GTGCTTGCGC GAGTTGGACA AGGTGTACGG ATTTGGCCTG

1081 AACCTGCCTG CCACCATCGC CACCTTCCGC GCCGGCTGCA TTCTTCAGGG CTACCTGCTG

1141 GGGCCCATGA CGAAGGCCTT CGAGGAGAAC CCGAGCCTGC CCAACCTGAT GGACGCTTTC

1201 ACCAAGGAGA TAGTGGCGGG CCTTGATGAC TGCCGCCAGA TTCTCGCCAA GCTCACAGTG

1261 AACACGGCAG TGTCACTGCC GGTCATGATG GCCTCACTGT CCTACATCAA CGCCATGTAC

1321 ACGGAGACTC TTCCGTACGG ACAGCTGGTG TCGTTGCAGC GCGACGTCTT TGGCCGCCAC

1381 GGCTACGAGC GCACGGACAA GGACGGCCGT GAGTCGTTTG AATGGCCCGC ACTGCAGTAG

**c**

1 ATGTTCCGCC ATGTTTCGGC TGCTTCGGCG AGCTCGCTCG TCGCCGCACG CTCCTTCTCC

61 AGCACCAACG TCTACCTGGA CAAGCGCATC AAGGTAAAGA ATGAGGTGGT AGACATGGAC

121 GGCGACGAGA TGACGCGCAT TATCTGGTCG TTCATCAAGG AAAAGCTGAT CCTGCCCTAC

181 GTGGATGTCC CGATCAACTA CTTTGACCTC AGCGTGACTA ACCGTGACGC GACGAACGAC

241 AAGGTCACGG TTGAAGCCGC CGAAGCGATC AAGAAGTGCA ACGTTGGCAT CAAGTGCGCC

301 ACCATCACCC CCGATGAGGC CCGCGTGAAG GAGTTCAACC TGAAGAAGAT GTGGAAGAGC

361 CCAAACGGTA CCATCCGGAA CATCCTTGGC GGCACGGTGT TCCGTGAGCC GATCATCGTC

421 TCCAACATCC CACGTATCGT CCCTCAGTGG CACAATCCCA TCGTGGTCGG CCGCCACGCC

481 TTCGGCGACC AGTACAAGGC GACGGATGCT GTTTTGAAGC CCGGCAAGCT GCAGCTCGTG

541 CACACACCCG CCGACGGCAG CGCGCCGACA ACGCTGGATG TATACGACTT CAAGGGCGAG

601 GGTGTCGGGC TGGCCATGTA CAACACGAAG GAGAGCATTG AGGGCTTTGC GAAGAGCTGC

661 TTCCAGTACG CGTTGATGCG CAAGTACCCA CTGGTTTTGA CGACGAAGAA CACCATCCTG

721 AAGAAGTACG ATGGCATGTT CCTGCAGACC TTCCAGCGCA TGTATGACGA GCAGTACAAG

781 GCCGACTTCG AGAAGGCCGG CATCACGTAC AACCACCGCC TCATCGATGA CCAGGTGGCG

841 CAGATGATCA AGGGCGAGGG CGGCTTCGTG TGGGCGTGCA AGAACTACGA TGGCGACGTG

901 CAGAGCGACA TCGTGGCGCA AGGCTTCGGC TCGCTGGGCC TCATGACATC TGTGCTGATG

961 TGCCCGGATG GCAAGACAAT CGAGGCCGAG GCCGCCCACG GCACGGTGAC GCGTCACTAC

1021 CGCCAGCATC AGCAGGGCAA GGAGACGAGC ACGAACTCCG TCGCCTCCAT CTACGCCTGG

1081 ACGCGCGGTC TCGCCCATCG TGGCAAGCTT GACGGCAACA GCGACCTCGT CAAGTTCTCG

1141 GAGACGCTGG AGAGGGTGGT CGTCAAGGCG ATTGAGGATG GCCACATGAC GAAGGACCTG

1201 GCCCTCTGCG TCTACGGCTC CAGCGGCGTC AAGCGTGAAC ATTACGAGAC GACGGAGCAG

1261 TTCCTCGACA GCGTCGATGC AGCCCTGAAG AAGGCGATGA GCGCGTAA

**d**

1 TGAATCCCTT TTCAAGATGA GCGATTATCT TTCGAAGTTG AAGGAGCACG TGCTGGAGAG

61 CACCGAAGTC AATGGATGCA CACCGAGCAT GGCCACTTCG ACTTTCAATG CCCCGTATGA

121 GGTTGCACGC AGGACCAAGG TGCTGGGAGC CACGGACAGC AGCCTGTTGA GCTTGCCTGC

181 GTGGAAGCGC TTGCAGTCCC TGTACGAGAA GCACGGCAAC GAGTCCATCC TTTCTCATTT

241 TGAGAACGAT CATCAGCGCT TTGGGCGGTA CTCGATTGAG GTTGGCCTGC ACAGCGAGGA

301 AAATTTCCTC TTCCTCGACT ACTCCAAGTC GCACATCAAC GACGAGATCA AGGATGCGTT

361 GGTTGCGCTG GCCGAGGAAC GTGGAGTGCG GGCGTTCGCC AAGGCTATGT TTGATGGGCA

421 GCGGGTGAAC TCTACTGAGA ACCGCGCCGT GTTGCATGTG GCGCTGCGCA ACCGCAGTAA

481 CCGCCCGATC ATCGTTGACG GGAAGGATGT GATGACCGAT GTGAACAATG TGCTTGCCCA

541 AATGAAGGAC TTCACCGAAA AGGTCCGCAG CGGAGAGTGG AAGGGTCAGA CGGGCAAGAG

601 CATTTCCAAC ATAGTCAACA TCGGGATTGG CGGCAGCGAC CTTGGCCCGG TCATGGTGAC

661 CGAGGCACTG AAGCCGTTCT CCAAGCGCGA CATGCACTGC TTTTTCGTGT CCAACGTCGA

721 TGGGACACAC ATGGCTGAGG TTCTGAAGCA GGTGAACCTG GAGGAGACCA TCTTTATCAT

781 TGCAAGCAAG ACGTTCACTA CACAAGAAAC GTTGACGAAT GCCATGTCTG CACGCAACGC

841 GCTCATGGAC TACCTCAAAG CAAACAACAT CTCGACGGAT GGCGCCGTTG CAAAGCATTT

901 TGTTGCCCTA TCGACCAACA CGGAAAAGGT TCGCGAGTTT GGCATTGATA CCGTCAACAT

961 GTTTGTGTTC TGGGACTGGG TCGGTGGTCG CTACTCTGTG TGGTCCGCCA TCGGTCTCTC

1021 CGTGATGCTT TCGATCGGCT ACGACAACTT TGTGGAGTTC CTGACTGGCG CGCACGTGAT

1081 GGATAACCAC TTTGCGTCTG CACCGACGGA GCAGAACCTG CCGATGATGC TGGCTTTGGT

1141 CGGCATCTGG TACAACAACT TTTTCGGCGC GGAGACAGAG GCGGTGCTGC CGTACGACCA

1201 GTACCTGTGG CGTCTGCCGG CCTACCTTCA GCAGCTCGAC ATGGAGAGCA ACGGCAAGGG

1261 CGTGACCAAG AAGTCTGGTG CAGTGGCTGT GCAGACGGGC CCCATTATCT TCGGTGAGGC

1321 CGGCACAAAT GGTCAACATG CATTCTACCA GCTCATTCAC CAGGGCACCA AGATCATCCC

1381 GTGCGATTTC ATTGGCTGCG TCCAAACACA GAACCGTGTG GGCGACCACC ACCGGATCCT

1441 GATGAGCAAC TTTTTCGCGC AGACGGAGGC GCTCATGGTA GGAAAGAGTG CGGAGGAGGT

1501 CCGCCAGGAG CTGGCCAAGT CTGGTATGTC GGATGAGGCC ATTCAGAGTA TGATTCCGCA

1561 CAAAACGTTT ACGGGAAACC GTCCCAGCAA CTCGATCCTG GTGAATGCTC TTACTCCGCG

1621 TGCGCTGGGT GCTATCATCG CCATGTACGA GCACAAGGTT CTCGTCCAGG GCGCGATCTG

1681 GGGCATCAAC AGCTATGACC AGTGGGGTGT GGAGCTTGGC AAGGTGCTTG CCAAGTCTAT

1741 CTTGCCGCAA CTCAAGTCCG GCAACATCGT CTCCGATCAC GACGGCTCTA CGAACGGCCT

1801 GATCAACATG TTCAACACGC GCGCACATCT GTGAAAAAGT CTCTTGATGC TACTATTTAG

1861 AGCTGCGAAG TGCATGTTCT CTTTCCTTCC TTTGTTGGCG ATTCAACGGA CAGGTAGCGA

1921 GGATCG

**e**

1 ATGTCGGAAG AGCAGTCTCA TGCTGATCAG GATGCCTACG TGGCTGACGT CGACGGCATT

61 CTGGACGTGC TCCGCGCGCA AGTGCTGGAG CGGAAGCCAG ATGACATTTT CCAGTTCATC

121 TCCAAGTCAG CGCTTAGTCT GCAGAAGGAT AGGGGCGCAG AATCGTGCGA TCGCATCAAC

181 TGTAAGGTCA AGGATGAACA GAAGAGTCGT GCGTTGACTA TTATCGTGTT CGGTGCCAGC

241 GGGGATCTGG CCAAGAAGAA GACATTTCCT GCCCTCTTCG ATCTGTACTG CGGTGGGCTC

301 CTCCCCCCGG AGGTCAACAT TATCGGCTAT GCTCGCACCA AGGTGGATGA TGTCGAGAAG

361 TGGAAGCATG AGACGCTCAT GAAGTATTTT TCGAACTTGT CGGAGCGCGG ATGCCACGCC

421 GAGGACTTCT TGAAACACAT CAGCTACTTT TGCGGAGCGT ACGATAGTGT GGACGACTTC

481 AAGCGTCTTG ACGCAGTGAT TCGCGAGAAG GAGAATGCCT TTAAGGGCCC TGAGAAGGGT

541 GGAAACCGTC TCTTCTACCT TGCTCTTCCC CCATCGGTAT TTGCAAGTGT CTGCGAGAGC

601 ATTCACAAGG GTGCAATGCC GCAGGAAGTG GGGGGATGGG TGCGGGTGAT CATCGAGAAG

661 CCCTTTGGCC GCGATACCAA GAGCTCTGCC GAGCTGTCCC AAGCGCTGGA GCCGTTCTTC

721 GACGAGTCGC AGCTGTACCG CATTGACCAC TACCTCGGGA AGGAGATGGT GCAGAACATC

781 ATCACGACAC GCTTCGCCAA TCGTATCTTC AGCGCTGTGT GGAATGCGAG CAACATCGCG

841 TGCGTGCAGA TCACGTTCAA GGAGACCATC GGCACTGAGG GCCGCGGCGG ATACTTCGAC

901 AGCATCGGCA TTATTCGCGA TGTCATGCAA AACCACCTCA CCCAGATTCT TGCCCTGCTG

961 GCCATGGAGA AGCCGCGGTC GCTGGACGCT GAGTGCATCC GTGACGAGAA GGTGTCGGTA

1021 CTGAAGTGCA TTGAGCCGAT AACCAAGGAG AATTGTGTTC TGGGTCAGTA CACTGCCTCC

1081 GCCGATGGTT CCATCCCTGG TTACCTTGAG GACGTGACCG TACCCGAGGG CAGCACTTGC

1141 CCCACATTTG CCGTGATGCG ACTCAACATC AACAACGACC GGTGGGCCGG CGTCCCTTTT

1201 ATTCTCAAGG CCGGCAAGGC TGTGGAACAG AAGTACGTGG CGATTCGCAT TCAGTTCAGG

1261 GATGAGGTCC ACCCCTACGG CGAGGCGACA CAACGCAACG AGCTCGTCAT CCGTGCCCAG

1321 CCGTCCGAGG CTATGTATGT CAAGATCACC ACAAAGGTTC CTGGCCTTAG TGGAGATTTG

1381 CGGCAGACGC ACCAGACAGA GCTGGACCTC ACATACCACA CTCGGTACGA TGTGCGCCTT

1441 CCAGACGCCT ACGAGAGCCT CATCAACGAC GCACTGCTGG GCAACTCGAC AAACTTTGTG

1501 CGTAAGGACG AGCTCGATGT GGCATGGCGC ATCTTCACGC CGCTCCTGCA CCAGATTGAC

1561 TCTGGAGAGA TCAAACCGAT CCCGTACCAG GCCGGCACGC GCGGGCCTAA GGAAGCGGAT

1621 GAATTCATCG CCAACAACGG CTTCAAGCAC CAGAAAGGCT ACCACTGGCT TCCCTCGAAT

1681 AAGCTGTGA

**f**

1 ATGTCTGAGC TCGTAAAGCT TGAAGTGGGC CATCAGGACT ATGCCTGGGG CAAGGATGCC

61 GCGTCCAGCT TCGTGGCAAA GATGAAGGGC TTGACGAACG ACAAGTCCGG CAAGATGTTT

121 GCGGAGTTGT GGGTAGGCAC GCACCCCAAC TGCCCGTCCA GGATCGCCGA CGGCGACGCG

181 CAGCTGCTCG AGGAGTTCCT GAAGCAGCCA GAGAATAAAA AGAAGTACTT TTCAGAAGCT

241 CACCAGGCCA CGACCTTCCG TGACACAGTG CCCTACCTGC TGAAGATTCT GTCGATTCGC

301 ACGGCGCTGT CCATCCAGGC GCACCCGTGC AAGAAGCTTG CCGAGAAGCT GCACGCAGCG

361 AGGCCGGATA AGTACAAGGA CCCGAACCAC AAGCCAGAGC TCATTTGCGC CTTGACCCCC

421 TTTGAGGCAC TCTGCTGCTT CCGACCGCTC GGCGCCATCA TCGCGTATCT GAAGCGCATC

481 CCGGAGCTGG CGGAGCTTGT GGGCGCCGAC GCGGTGCTGG GTCAGTACAT GATGGCGCCG

541 GAGAGCGCGC TGCCTGCAAC GGACAGCGAC GAAGAGAAGC AGTCGCTGAA AGCGATGATA

601 ACGAACGTGT ACGCTGCTTC GGACGACATC GTCACGAAGG CGCTGCGCCT GCACCTCCAG

661 CGCATCGAGG AGACGGGCGC GCAGTGTGCC GAGGACGAGC TCTTTGTTCG CATTTACAGG

721 CAGTACCCGG ATGATGTCGG CTGCTGGATG GTTTACTTCC TCAATTACGT ACAGATGGTG

781 CCCGGGGAGG CTCTCTTCTT ATCGGACAGC GAGCCGCACG CGTATATCAG CGGCGACGGT

841 GTCGAGATCA TGGCATGCAG CGACAACGTC GTGCGTGCTG GACTCACGCC GAAGTGGAAG

901 GATGTGCCGA CGCTCATAAG CATGCTGAAG TACGACACGA CCGGGCTTGC GTCTGCCCGC

961 CACGAGAAGA AGAGCAGCGA GGACGCGGCG CAGTGGCAGG TGCAGTACTA CCGGCCACCG

1021 GCACAGTTCC CGGACTTTTC GCTGTACCGC ATGCAGTACG AGCACGCTTC TGGCAAAGGG

1081 ACGACCTCCG TGACCTTGCC GACGATAGGC CTGGGCTTCT GCTTGGAGGG GTCTGCCAAG

1141 GTGAACGGGA CGACGGTAAA CGCCGGCGAC TGCTTTGCGG TGCCGTACGG CAAGGTGACG

1201 TGCCAAGCTG AGGGAGCGAA GGCGCTTGTG TTCGTTGCGT CGACCAACGA CTTGAGCGAC

1261 AGGTAG

**g**

1 ATTTACGAGG AGACGGTTAA GATTACGCAC ATCAAGATGG CCGCGACGCT GCCGGAGGTG

61 GATATCCACA CCCTCGGCAC CTACACCTTT GACGACTACA ACTTCCAGGT GGAGGTGGTT

121 GACAGCTTGG CTGACTACGC TGCGTACATG CAGGAGGTGT TCGACTTCGA GGCCATCAAG

181 GCACTCGTGC AGCGCCTCGA CTTCAAGGTC CACGTGGACA GCCTTCACGG CGTCAGCGGC

241 CCGTACGTTG ATCGCATCTT TCACGAATGC CTTGGTGTGC CCAAGGCCTC CCTATTCCGC

301 ACGAACGTCC TGCCCGACTT TGGCGGCTGC CACCCCGATC CGAATCTCAC GTACGCGGCC

361 GACTTGGTGC ATGTGATGGG GCTGCTGCCA GACGGCAATG CGAACCCCGC GATGAAGCAT

421 ATCAGCACGG TGCCCAGCTT CGGTGT
