# Supplementary material for: Exploiting genetic polymorphisms in metabolic enzymes for rapid screening of Leishmania infantum genotypes
Source: Parasit Vectors. 2018 Nov 1;11:572. doi: 10.1186/s13071-018-3143-7 (PMC6211443; doi:10.1186/s13071-018-3143-7)
Supplement: Supplementary file 2 — Table S1. Summary of allelic profile and genotype of L. infantum zymodemes considered in this study. (DOCX 18 kb) [file 13071_2018_3143_MOESM2_ESM.docx]

**Table S1.** Summary of allelic profile and genotype of *L. infantum* zymodemes considered in this study

| **Zymodeme** | **Strain/isolate** | ***me*** | ***pgd*** | ***icd*** | ***gpi*** | ***g6pdh*** | ***mpi*** | **Genotype** |
| --- | --- | --- | --- | --- | --- | --- | --- | --- |
| MON-1 | MHOM/FR/78/LEM75 | 1 | 1 | 1 | 1 | 1 | 1 | 1 |
| MON-1 | MHOM/ES/1993/PM1 | 1 | 1 | 1 | 1 | 1 | 1 | 1 |
| MON-1 | MHOM/FR/1995/LPN114 | 1 | 1 | 1 | 1 | 1 | 1 | 1 |
| MON-1 | MHOM/PT/2000/IMT260 | 1 | 1 | 1 | 1 | 1 | 1 | 1 |
| MON-1 | MHOM/FR/1997/LSL29 | 1 | 1 | 1 | 1 | 1* | 1 | 1* |
| MON-1 | MHOM/ES/1986/BCN16 | 1 | 1 | 1 | 1 | 1 | 1 | 1 |
| **MON-1** | **MHOM/TN/80/IPT1** | **2** | **1** | **1** | **2** | **1** | **1** | **2** |
| **MON-1** | **MHOM/FR/78/LEM75** | **1** | **1** | **1** | **1** | **1** | **1** | **1** |
| **MON-1** | **Isolate 31U** | **1** | **1** | **1** | **1** | **1** | **1** | **1** |
| **MON-1** | **Isolate 49u** | **1** | **1** | **1** | **1** | **1** | **1** | **1** |
| **MON-1** | **Isolate 10816** | **1** | **1** | **1** | **1** | **1** | **1** | **1** |
| **MON-1** | **Isolate 791** | **1** | **1** | **1** | **1** | **1** | **1** | **1** |
| **MON-1** | **Isolate V2921** | **1** | **1** | **1** | **1** | **1** | **1** | **1** |
| MON-1 | MHOM/BL/67/ITMAP263 | 1 | n.a. | n.a. | n.a. | 1 | n.a. |  |
| MON-11 | MHOM/FR/1980/LEM189 | 2 | 2 | 2 | n.a. | 2 | 1 |  |
| **MON-24** | **MHOM/DZ/82/LIPA59** | **3** | **2** | **3** | **1** | **3** | **1** | **3** |
| MON-24 | IARI/PT/1989/IMT171 | n.a. | 2 | n.a. | n.a. | n.a. | n.a. |  |
| MON-27 | MHOM/IT/1979/FRANCESCA | n.a. | 1 | n.a. | 1* | n.a. | n.a. |  |
| **MON-29** | **MHOM/ES/82/BCN1** | **4** | **2** | **1** | **1** | **2** | **1** | **4** |
| MON-29 | MHOM/FR/1996/LEM3249 | 2 | 2 | 2 | 1 | 4 | 1 | 5 |
| MON-34 | MHOM/CN/1980/A | n.a. | **2** | n.a. | **2** | n.a. | n.a. |  |
| **MON-72** | **MHOM/IT/86/ISS218** | **1** | **1** | **1** | **1** | **1** | **1** | **1** |
| MON-77 | MCAM/ES/86/LEM935 | n.a. | n.a. | n.a. | n.a. | 1* | n.a. |  |
| MON-78 | MHOM/MT/1985/BUCK | 5 | 1 | 2 | 2 | 3 | 1 | 6 |
| MON-81 | MHOM/SD/1962/3S^a^ | 6 | 3 | 4 | 3 | 5 | 1 | 7 |
| MON-98 | MHOM/GR/2001/GH6 | 2 | n.a. | n.a. | n.a. | n.a. | n.a. |  |
| MON-98 | MCAN/GR/2001/GD8 | 2 | n.a. | n.a. | n.a. | n.a. | n.a. |  |
| MON-98 | MHOM/GR/2003/GH15 | 2 | n.a. | n.a. | n.a. | n.a. | n.a. |  |
| MON-98 | MHOM/GR/2003/GH16 | 2 | n.a. | n.a. | n.a. | n.a. | n.a. |  |
| MON-98 | MHOM/GR/2003/GH18 | 2 | n.a. | n.a. | n.a. | n.a. | n.a. |  |
| MON-98 | MHOM/GR/2003/GH20 | 2 | n.a. | n.a. | n.a. | n.a. | n.a. |  |
| MON-98 | MHOM/GR/2004/GD17 | 2 | n.a. | n.a. | n.a. | n.a. | n.a. |  |
| MON-105 | MHOM/ES/2001/LLM1026 | n.a. | n.a. | n.a. | n.a. | 1 | n.a. |  |
| MON-136 | MHOM/IT/1990/ISS510 | n.a. | n.a. | n.a. | n.a. | n.a. | 2 |  |
| MON-183 | MHOM/ES/1991/LEM2298 | 2 | 2 | 1 | 4 | 1* | 1 | **8** |
| MON-188 | MHOM/IT/1993/ISS800 | 7 | 2 | 2 | n.a. | 3 | 3 |  |
| MON-198 | MHOM/ES/1988/LLM175 | 2 | 2 | 1 | 4 | 2 | 1 | 9 |
| MON-199 | MHOM/ES/1992/LLM373 | 8 | 2 | 1 | 4 | 1* | 1 | 10 |
| **MON-201** | **MHOM/IT/93/ISS822** | **1** | **1** | **1** | **1** | **1** | **1*** | **1*** |
| MON-228 | MHOM/IT/1994/ISS1036 | 2* | 2 | 2 | 1* | 1 | 1 | 11 |
| MON-253 | MHOM/ES/1996/LLM580 | n.a. | n.a. | n.a. | n.a. | 6 | n.a. |  |
| MON-267 | MHOM/SD/1997/LEM3472^a^ | 9 | 3 | 5 | 3 | 5 | 4* | 12 |
| MON-267 | MCAN/SD/2000/LEM3988 | n.a. | n.a. | n.a. | n.a. | 7 | n.a. |  |
| MON-309 | ITOB/TR/2005/CUK2 | 7* | n.a. | 2 | n.a. | 3 | 1 |  |
| MON-309 | MHOM/TR/2005/CUK1 | 7 | n.a. | 2 | n.a. | 3 | 1 |  |
| NI | ITOB/TR/2007/CUK10 | 7* | n.a. | 2 | n.a. | 3 | 1 |  |
| NI | MHOM/CN/93/KXG-XU | n.a. | n.a. | n.a. | n.a. | 8 | 4 |  |
| NI | MHOM/CN/94/KXG-LIU | n.a. | n.a. | n.a. | n.a. | 8 | 4 |  |
| NI | MHOM/PA/78/WR285 | n.a. | n.a. | n.a. | n.a. | 1 | n.a. |  |
| NI | Isolate D38 | n.a. | n.a. | n.a. | n.a. | 1 | n.a. |  |
| NI | Isolate D33 | n.a. | n.a. | n.a. | n.a. | 1 | n.a. |  |
| NI | Isolate D36D | n.a. | n.a. | n.a. | n.a. | 1 | n.a. |  |
| NI | Isolate E9D | n.a. | n.a. | n.a. | n.a. | 1 | n.a. |  |
| NI | Isolate RRR-B | 2 | n.a. | n.a. | n.a. | 1 | 1* |  |
| non-MON-1 | MHOM/TR/2000/OG-VL | 2 | n.a. | 1 | n.a. | 1 | 1 |  |

* Heterozygosity

^a^initially assigned to *L. infantum* and successively designated *L. donovani*

NI not indicated

n.a. not available
