# Supplementary figures and images for: Exploiting genetic polymorphisms in metabolic enzymes for rapid screening of Leishmania infantum genotypes
Source: Parasit Vectors. 2018 Nov 1;11:572. doi: 10.1186/s13071-018-3143-7 (PMC6211443; doi:10.1186/s13071-018-3143-7)

Reference strains

MHOM/TN/1980/IPT1

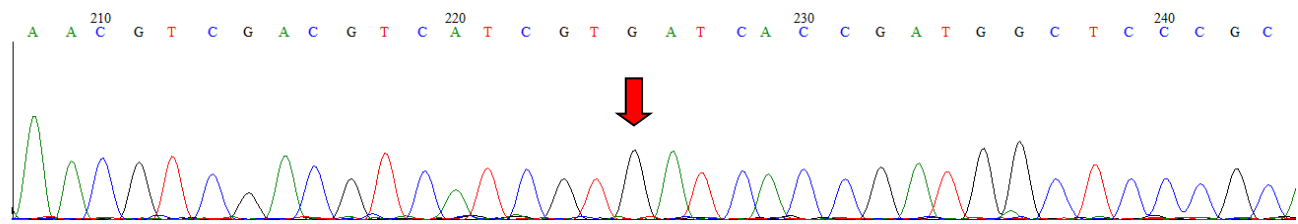

MHOM/FR/78/LEM75

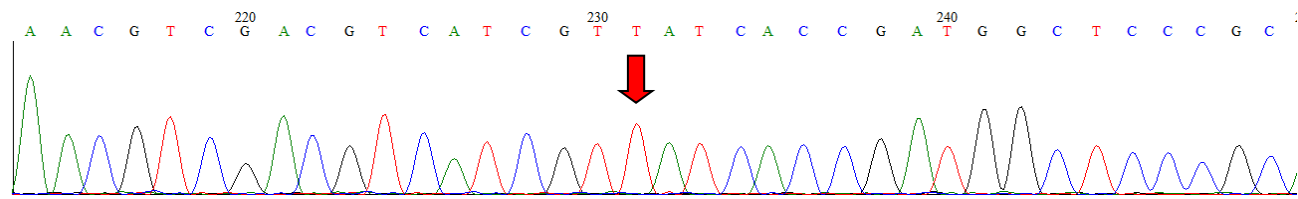

MHOM/DZ/82/LIPA59

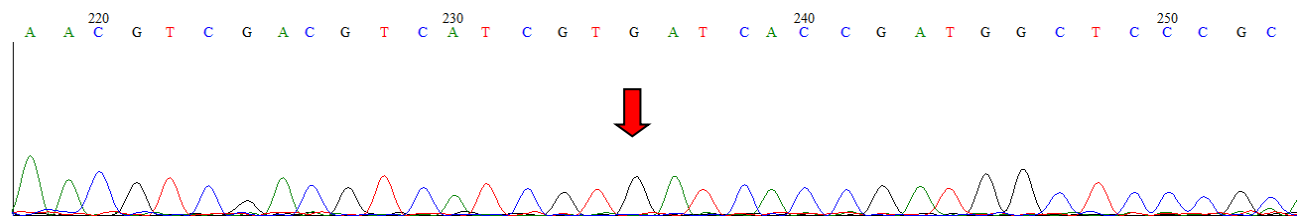

Clinical isolates

Isolate 1

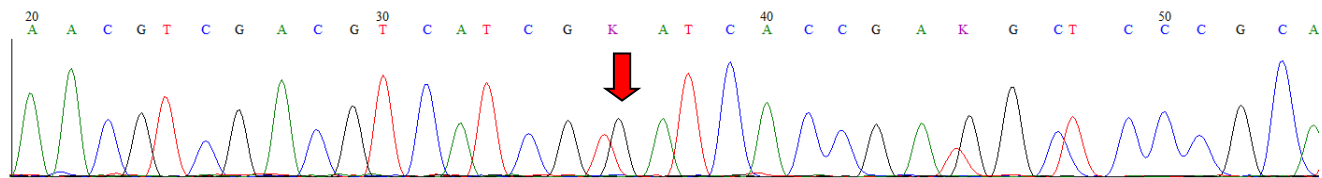

Isolate 2

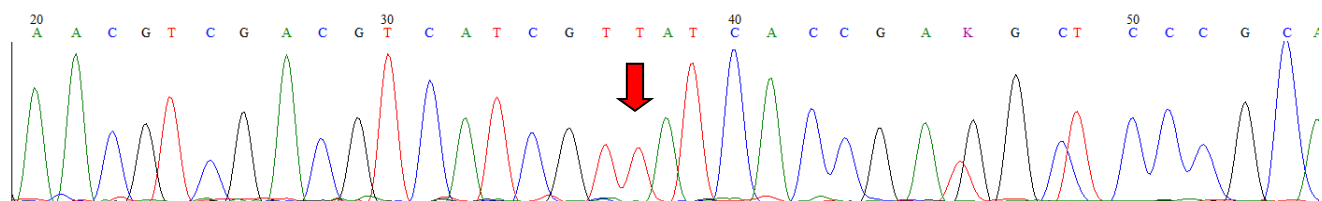

Supplement: Supplementary file 3 — Figure S2. Partial sequences of qPCR-MEint amplification products from reference strains and clinical isolates. Electropherograms encompassing polymorphic nucleotide at position 390 (arrows) are represented. (PDF 72 kb) [file 13071_2018_3143_MOESM3_ESM.pdf]

Clinical samples

77

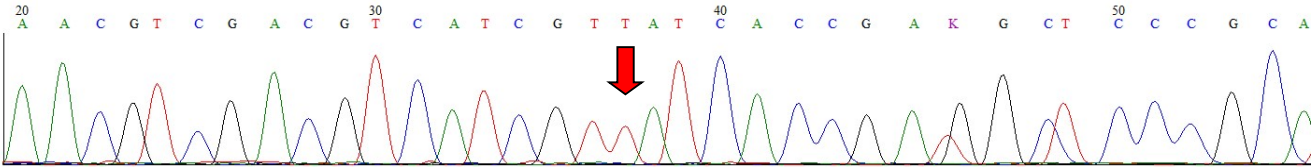

62

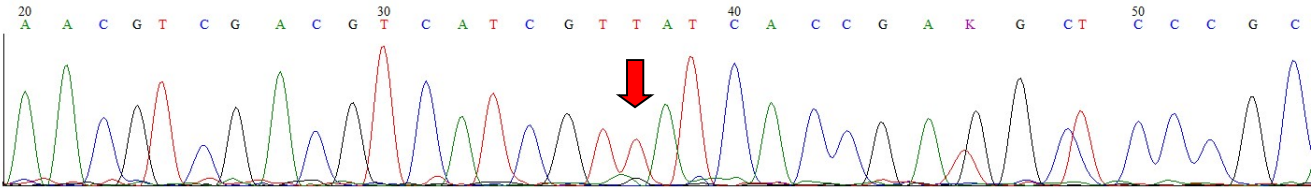

psalb

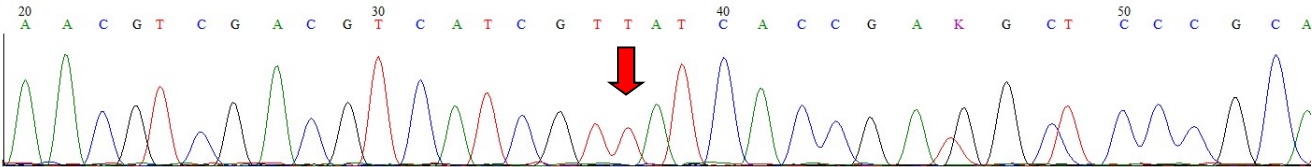

Vea

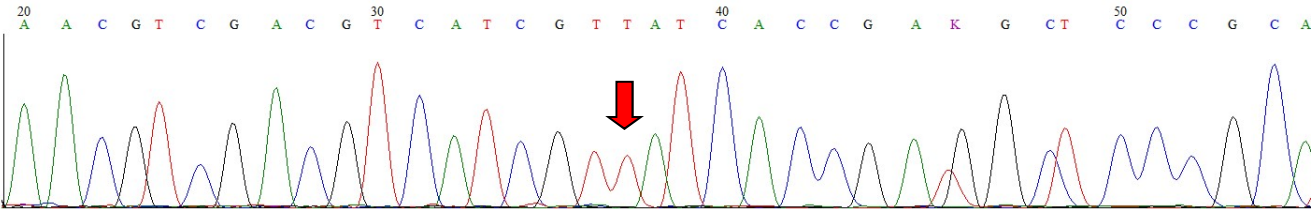

Supplement: Supplementary file 4 — Figure S3. Partial sequences of qPCR-MEint amplification products from clinical samples. Electropherograms encompassing polymorphic nucleotide at position 390 (arrows) are represented. (PDF 570 kb) [file 13071_2018_3143_MOESM4_ESM.pdf]
